# Special Care Is Needed in Applying Phylogenetic Comparative Methods to Gene Trees with Speciation and Duplication Nodes

**DOI:** 10.1093/molbev/msaa288

**Published:** 2020-11-10

**Authors:** Tina Begum, Marc Robinson-Rechavi

**Affiliations:** 1 Department of Ecology and Evolution, University of Lausanne, Lausanne, Switzerland; 2 SIB Swiss Institute of Bioinformatics, Lausanne, Switzerland

**Keywords:** ortholog, paralog, gene expression, phylogenetic comparative methods, Brownian, Ornstein–Uhlenbeck

## Abstract

How gene function evolves is a central question of evolutionary biology. It can be investigated by comparing functional genomics results between species and between genes. Most comparative studies of functional genomics have used pairwise comparisons. Yet it has been shown that this can provide biased results, as genes, like species, are phylogenetically related. Phylogenetic comparative methods should be used to correct for this, but they depend on strong assumptions, including unbiased tree estimates relative to the hypothesis being tested. Such methods have recently been used to test the “ortholog conjecture,” the hypothesis that functional evolution is faster in paralogs than in orthologs. Although pairwise comparisons of tissue specificity (τ) provided support for the ortholog conjecture, phylogenetic independent contrasts did not. Our reanalysis on the same gene trees identified problems with the time calibration of duplication nodes. We find that the gene trees used suffer from important biases, due to the inclusion of trees with no duplication nodes, to the relative age of speciations and duplications, to systematic differences in branch lengths, and to non-Brownian motion of tissue specificity on many trees. We find that incorrect implementation of phylogenetic method in empirical gene trees with duplications can be problematic. Controlling for biases allows successful use of phylogenetic methods to study the evolution of gene function and provides some support for the ortholog conjecture using three different phylogenetic approaches.

## Introduction

The “ortholog conjecture,” a standard model of phylogenomics, has become a topic of debate in recent years (Koonin 2005; Studer and Robinson-Rechavi 2009; Nehrt et al. 2011; Altenhoff et al. 2012; Chen and Zhang 2012; Gabaldón and Koonin 2013; Rogozin et al. 2014; Kryuchkova-Mostacci and Robinson-Rechavi 2016; Dunn et al. 2018; Stamboulian et al. 2020). The ortholog conjecture is routinely used by both experimental and computational biologists in predicting or understanding gene function. According to this model, orthologs (i.e., homologous genes which diverged by a speciation event) retain equivalent or very similar functions, whereas paralogs (i.e., homologous genes which diverged by a duplication event) share less similar functions (Studer and Robinson-Rechavi 2009). This is linked to the hypothesis that paralogs evolve more rapidly. This hypothesis was challenged by results suggesting that paralogs would be functionally more similar than orthologs (Nehrt et al. 2011). Such findings not only raised questions on the evolutionary role of gene duplication but also questioned the reliability of using orthologs to annotate unknown gene functions in different species (Sonnhammer et al. 2014). Several studies (Altenhoff et al. 2012; Chen and Zhang 2012; Rogozin et al. 2014; Kryuchkova-Mostacci and Robinson-Rechavi 2016) later found support for the ortholog conjecture, mostly based on comparisons of gene expression data. 

Although all previous studies of the ortholog conjecture had used pairwise comparisons of orthologs and paralogs, a recent article suggested that this was flawed, and that phylogenetic comparative methods should be used (Dunn et al. 2018). Phylogenetic structure can violate the fundamental assumption of independent observations in statistics, and thus ignoring it can lead to mistakes (Felsenstein 1985). A solution is to use phylogeny-based methods. Phylogenetic independent contrast (PIC) (Felsenstein 1985) and phylogenetic generalized least square (Martins and Hansen 1997; Grafen 1989; Rohlf 2001) are the most commonly used phylogenetic comparative methods. They were developed under a purely neutral model of evolution, that is, Brownian motion (BM). Such Brownian processes have been extended under maximum likelihood, to allow different rates of evolution on different branches of a phylogeny (O’Meara et al. 2006; Thomas et al. 2006) and to include stabilizing selection in which the trait is shifted toward a single fitness optimum, or multiple different adaptive optima (i.e., “Ornstein–Uhlenbeck” or OU process) (Hansen 1997; Butler and King 2004; Beaulieu et al. 2012). Such phylogenetic modeling requires a priori knowledge of different states on the tree. Alternatively, Markov chain Monte Carlo (MCMC) sampling in a Bayesian framework has been used to accurately estimate the number, location, and magnitude of shifts in evolutionary rates, or in optimal trait values without a priori assignment of states (Eastman et al. 2011; Pennell et al. 2014; Uyeda and Harmon 2014; Catalán et al. 2019). Bayesian approaches are time consuming, whereas OU modeling with phylogenetic lasso algorithm allows a faster detection of shifts in optimal trait value (Khabbazian et al. 2016). Moreover, OU has been used to model gene expression evolution (Rohlfs and Nielsen 2015; Chen et al. 2019).

Among phylogenetic methods, PIC is widely adopted for its relative simplicity and its applicability to a wide range of statistical procedures (Cooper, Thomas, FitzJohn, et al. 2016; Dunn et al. 2018). The performance of PIC relies on three basic assumptions: a correct tree topology, accurate branch lengths, and trait evolution following BM (where trait variance accrues as a linear function of time) (Felsenstein 1985; Garland 1992; Garland et al. 1992; Díaz-Uriarte and Garland 1998; Freckleton and Harvey 2006; Cooper, Thomas, FitzJohn, et al. 2016). If any of these assumptions is incorrect, this can lead to incorrect interpretation of results, unless biases are controlled for (Díaz-Uriarte and Garland 1996, 1998). Although previous applications of PIC studied multivariate traits on pure speciation trees, Dunn et al. (2018) took an innovative approach in applying PIC to compare the divergence rates of a univariate trait between two different node events (“speciation” and “duplication”), to test the ortholog conjecture. They performed extensive analyses in support of their results. However, such an application might be problematic because the time of occurrence of gene duplication, one of the two types of events compared, is unknowable by external information (e.g., no fossil evidence). Therefore, further study is required to understand why Dunn et al. (2018) obtained results which are inconsistent with previous studies. It is possible that all the conclusions drawn by previous studies on gene duplication are incorrect due to overlooking phylogenetic tree structure. If so, it should be well supported.

We re-examined the data of Dunn et al. (2018), after reproducing their results using the resources and scripts provided by the authors. We have uncovered problems with the use of PIC on biased calibrated gene trees, violation of the underlying assumptions, and the inclusion of pure speciation gene trees. We used PIC on gene trees after fixing the calibration bias for old duplication nodes. With proper controls, the phylogenetic method supports the ortholog conjecture. To verify this result, we also applied data modeling approaches using a maximum likelihood framework, and using a reversible-jump Bayesian MCMC algorithm. Support for the ortholog conjecture still holds with proper controls.

## Results

### Issues with Straightforward Application of PICs

Dunn et al. (2018) have made a relevant argument that the ortholog conjecture test should be done in a phylogenetic framework, as closely related species or genes tend to share more similar traits. They applied PIC to 8,520 time-calibrated trees (table 1) and reported evidence against the ortholog conjecture for tissue specificity τ (median: PIC_speciation_ = 0.0072, PIC_duplication_ = 0.0051, one-sided Wilcoxon test *P *=* *1). Yet, the same data supported the ortholog conjecture when analyzed by pairwise comparisons both in Kryuchkova-Mostacci and Robinson-Rechavi (2016) and in the re-analysis by Dunn et al. (2018). To understand the incongruence between PIC and pairwise comparisons, they performed simulations of τ on their trees under the ortholog conjecture and under a null of uniform BM. Both methods should be able to distinguish the null from the ortholog conjecture for diverse trees (supplementary fig. S1, Supplementary Material online). Under the simulations of Dunn et al. (2018), pairwise comparisons could not distinguish the two scenarios, whereas the PIC could. As their results on empirical data resembled those on the null simulation, they questioned both the use of pairwise comparisons and the support for the ortholog conjecture from tissue specificity data.

**Table 1.** Information on Different Tree Sets, Number of Internal Node Events, and Node Ages Used in This Reanalysis.

Data Sets	Number of Trees	Number of Speciation Events	Number of Duplication Events	Number of NA Events	Maximum Speciation Node Age (My)	Maximum Duplication Node Age (My)
Dunn et al.: full set	8,520	67,911	21,071	26,794	296	11,799,977
Dunn et al.: trees with strong phylogenetic signals	2,082	13,118	4,056	5,186	296	1,342
This study: after excluding pure speciation trees	4,288	38,882	15,274 (8,556 young + 6,718 old)	15,201	296	1,175

Note.—My, million years; young, age ≤ 296 My; old, age > 296 My.

**Fig. 1.**
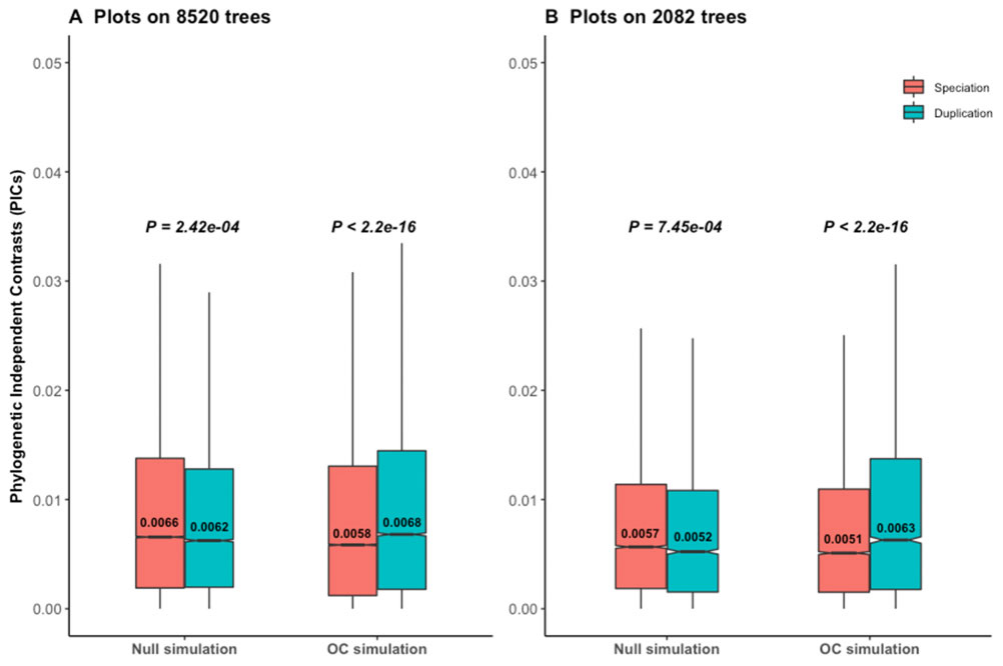
Reanalyses of phylogenetic simulation data of Dunn et al. (2018). *P* values are from Wilcoxon two-tailed tests. Values inside boxplots denote median PIC values of the corresponding events. In null simulations, there should be no difference in contrasts between events. In OC (ortholog conjecture) simulations, contrasts are expected to be higher for duplication than for speciation. (*A*) Higher contrasts for speciation than duplication reject the null hypothesis under null simulation scenario for all empirical time calibrated gene trees. (*B*) Results are similar with a subset of trees with strong phylogenetic signal for τ.

To understand their results, we first reproduced and reanalyzed the data of Dunn et al. (2018), focusing on the phylogenetic approach. Dunn et al. reported a nonsignificant result (*P *=* *1) for the PIC under the null simulation as well as for the empirical data, using a Wilcoxon one-tailed rank test to check whether the contrasts of duplication events are higher than the contrasts of speciation events. Surprisingly, our reanalysis with a Wilcoxon two-tailed rank test on the same data shows that the PIC rejects the null hypothesis on the null simulations (fig. 1*A*), with significant support for higher contrasts after speciation than duplication. This means that the PIC method supports a trend opposite to the trend expected under the ortholog conjecture, in a null simulation. This was robust to repeating the simulations with different random seed number (supplementary fig. S2, Supplementary Material online). This indicates that neither of the approaches, PIC or pairwise, worked properly for these calibrated trees, as both the approaches reject the null hypothesis when simulations are performed under the null. Moreover, when we used a Wilcoxon two-tailed rank test instead of a one-tailed test on the empirical data, the result was also significant (*P *<* *2.2e^−16^), in the same unexpected direction.

Statistical nonindependence among species trait values because of their phylogenetic relatedness can be measured by phylogenetic signal (Pagel 1999; Freckleton et al. 2002; Blomberg et al. 2003; Münkemüller et al. 2012; Molina-Venegas and Rodríguez 2017). Use of the PIC is mainly important for the data sets with strong phylogenetic signal, where it allows to recover phylogenetically independence. Dunn et al. (2018) used Blomberg’s *K*. Its value ranges from 0 to ∞ for each tree, where a value of 0 indicates no phylogenetic signal for the trait studied, and a value close to 1 or higher indicates strong phylogenetic signal (Pagel 1999; Freckleton et al. 2002; Blomberg et al. 2003; Münkemüller et al. 2012; Molina-Venegas and Rodríguez 2017). With a cutoff of *K* > 0.551, Dunn et al. (2018) obtained only 2,082 trees (table 1), 24.4% of the total, with strong phylogenetic signal. The phylogenetic method still rejects the null hypothesis under null simulations for those 2,082 trees using a Wilcoxon two-tailed rank test (fig. 1*B*), showing that the problem is not simply due to low phylogenetic signal. Using a cutoff of *P *<* *0.05 together with *K* > 0.551 leads to 1,135 statistically significant trees with strong phylogenetic signals, for which we obtained a similar result (supplementary fig. S3, Supplementary Material online). This means that the bias is not limited to the selection of tree sets, or to the number of speciation or duplication events used for the analyses. As the trend was similar for these 1,135 trees, we continued analyses with the 2,082 trees of Dunn et al. (2018) for consistency.

The accuracy and performance of the PIC method largely depend on proper branch length calibration in absolute time (e.g., in million years—My) (Garland 1992; Díaz-Uriarte and Garland 1998; Cooper, Thomas, FitzJohn, et al. 2016). We thus investigated possible biases created during calibration of gene trees. Due to nonavailability of external references for duplication time points (e.g., no fossils), Dunn et al. (2018) used only seven speciation time points to calibrate substitution rate trees. The ages of other node events are estimated using penalized likelihood (Sanderson 2002) and vary for the same duplication clade labels even within the same gene trees. The oldest speciation age for their calibrated trees was 296 My (table 1), corresponding to the use of chicken as the outgroup. Surprisingly, the calibrated node age of the oldest duplication event was 11,799,977 My (table 1 and supplementary table S1, Supplementary Material online), that is, 2,600 times older than the Earth. This is indicative of issues with time calibration. It is due to the fact that pruning was done prior to the time calibration in Dunn et al. (2018). Pruning to species with τ data leads to trees with many duplication (or NA) nodes older than 296 My. If there were older (>296 My) speciation events before pruning, they are also removed (supplementary fig. S4*A*, Supplementary Material online). If the root node of a pruned tree is a speciation, the duplication ages are constrained by this speciation. Otherwise, there are no constraints for the duplication events older than the oldest speciation events (supplementary fig. S4 and table S1, Supplementary Material online), which can introduce a calibration bias. This unreliable branch length estimation for the old duplication nodes eventually led to much larger expected variances for gene duplication events than for speciation events (supplementary fig. S5*A* and *B*, Supplementary Material online).

PIC of a node is a ratio of changes in trait values (τ here) for descendant nodes to their expected variance, that is, the lengths of the two branches that connect the node to its two descendants. In this study, lower contrasts refer to lower PIC values, whereas the lower contrasts variance means lower calibrated branch lengths. This means that similar changes in τ for two nodes can produce different PIC values, with the lower contrast for the node with higher expected variance (i.e., calibrated branch length). In the null simulations only the τ values are simulated, whereas the branch lengths (hence the expected variances) are taken from the empirical data, and thus share its biases. This explains why contrasts are lower for duplications than for speciations under null simulations as well as with empirical data. Such calibration bias in branch lengths violates the second assumption of PIC applicability and inflates type I error rates (Díaz-Uriarte and Garland 1996, 1998).

### Randomization Tests to Assess the Performance of Phylogenetic Method

**Fig. 2.**
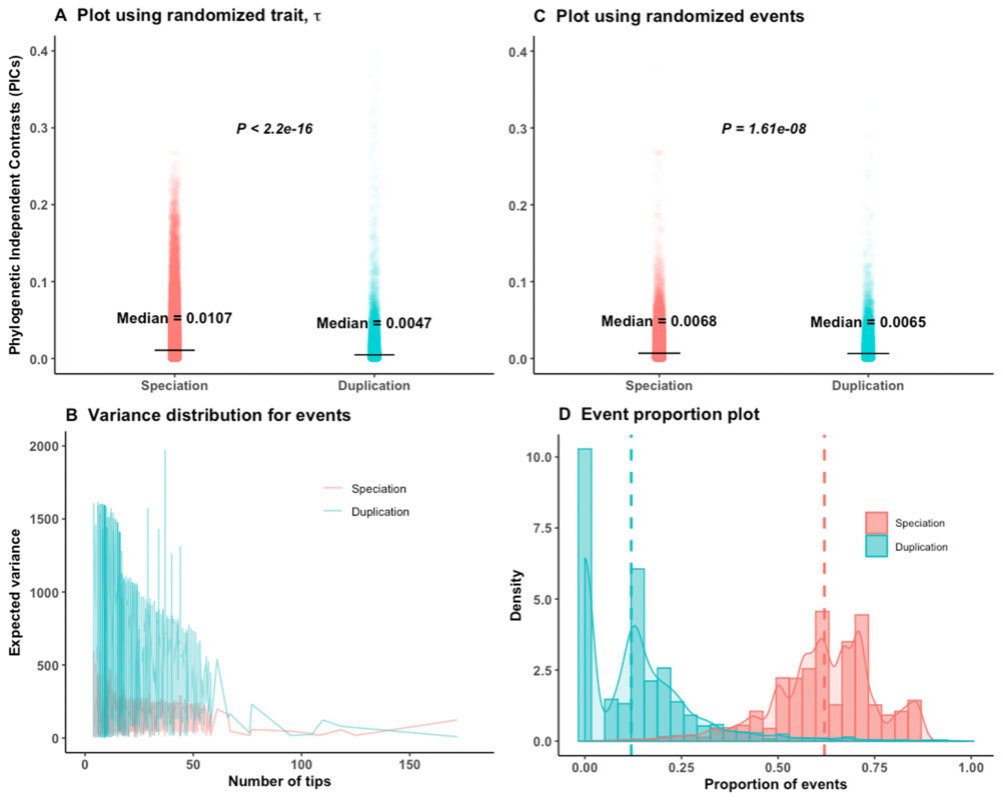
Analyses on calibrated empirical gene trees of Dunn et al. (2018). *P* values are from Wilcoxon two-tailed tests. (*A*) Randomly shuffling the τ values of the tips for 8,520 gene trees does not alter the empirical trend of an opposite trend to the ortholog conjecture. (*B*) The expected variance is much higher for duplication than speciation events irrespective of the number of tips considered for the study. (*C*) Using the original τ data, if we permute the events (Speciation or Duplication or NA) of the nodes, the trend of result remains. (*D*) The proportion of speciation events is much higher than duplication events for all time-calibrated trees; the dotted line represents the median proportion of both events; a high proportion of trees have no duplication events.

We used randomization tests to assess bias in different analyses of the empirical data set. Our expectation is that the trend of the empirical result should differ from the randomized ones. In a first randomization test, we permuted the τ values across the tips of each tree without altering the node events of the trees. By such randomization, the real phylogenetic relationships between trait values are removed for each tree. When we compared the node contrasts of the speciation and duplication events computed based on these 8,420 randomized τ trees (fig. 2*A*), we found the same pattern as reported for the empirical gene trees by Dunn et al. (2018), contrary to expectation. It confirms that results are driven by their large differences in branch lengths (i.e., in expected variances) (fig. 2*B*), as on simulated null data. Any effect of trait divergence rates of speciation and duplication events is always masked by this branch length difference of node events. This violates the basic assumption of applicability of the PIC method to Brownian trait evolution. To remove the problem of difference in expected variances of the two events, we performed a second randomization test: We kept the original τ value for tips but randomly shuffled the events (duplication, speciation, or NA) of internal nodes of the 8,420 empirical gene trees to maintain the original proportions of speciation and duplication events. The resulting trend (fig. 2*C*) still resembled the empirical gene trees data. This appears due to the fact that the majority of the nodes are speciations (fig. 2*D* and table 1) with node ages ≤296 My. Most of the trees with many duplication events on the other hand have ancient duplication events for which the evolutionary rates of duplication are often masked by the effect of longer branch lengths. Opposite to our expectation, the calibrated trees with no or few duplications have higher overall nodes contrast (apparent fast evolution) than trees with many duplications (apparent slow evolution). This might be due to greater difficulty in detecting paralogs for fast-evolving genes. Therefore, reshuffling of events may not change the observed pattern of higher speciation contrasts than duplication contrasts.

Out of 8,520 calibrated trees, 2,990 were pure speciation trees with no duplication events. For these 2,990 trees, random shuffling of events had no impact. To avoid this bias, we removed those 2,990 speciation trees as well as trees with negative branch lengths and randomized the trait or the internal node events 100 times on the remaining 5,479 trees. However, we still always obtained significantly higher contrasts of speciation than of duplication (supplementary fig. S6*A* and *B*, Supplementary Material online). The randomization pattern is the same restricting to 2,082 trees with strong phylogenetic signal (supplementary fig. S6*C* and *D*, Supplementary Material online).

All these analyses indicate that the results reported by Dunn et al. (2018) are biased by the calibrated phylogeny structures and that this bias is not easy to correct. We propose three approaches to correct for this bias and recover a proper phylogenetic signal of trait evolution.

### Approach 1: PIC with Diagnostic Tests

Diagnostic tests for each tree are essential to ensure phylogenetic independence of node contrasts, especially as there is evidence of bias in the calibrated trees. This can be verified by absence of correlation between the absolute value of PICs and their standard deviations, node height, node age, or node depth (Garland 1992; Garland et al. 1992; Díaz-Uriarte and Garland 1996, 1998; Freckleton 2000; Freckleton and Harvey 2006; Cooper, Thomas, FitzJohn, et al. 2016). A statistically significant negative or positive correlation in any of the diagnostic tests confirms that the PICs for that tree are nonindependent (Garland 1992; Garland et al. 1992; Díaz-Uriarte and Garland 1996, 1998; Freckleton 2000; Freckleton and Harvey 2006; Cooper, Thomas, FitzJohn, et al. 2016); in practice, we used *P *<* *0.05 for significance.

**Fig. 3.**
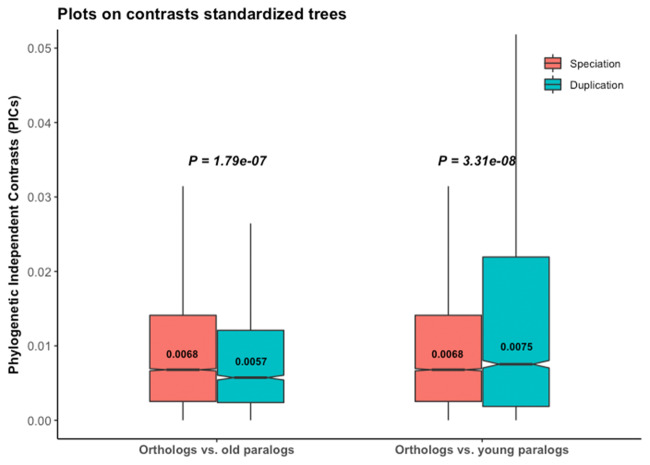
The ortholog conjecture test on τ for trees passing diagnostic plot tests. *P* values are from Wilcoxon two-tailed tests. Values inside boxplots denote median PIC values of the corresponding events. Young duplicates: age ≤ 296 My, the maximum speciation age; old duplicates: age >296 My.

We performed such diagnostic tests on 4,288 trees, for which calibration biases are fixed for old duplication nodes (see Materials and Methods, table 1). Among them only 2,088 (48.7%), which includes 15,321 speciation and 6,213 duplication nodes, passed all four diagnostics tests for τ evolution. We performed our PIC analyses separately for 3,948 young (≤296 My, the oldest speciation in the trees) and 2,265 old (>296 My) duplication events. Analyses on young duplicates after diagnostic tests provided support for the ortholog conjecture (fig. 3), but old duplicates did not. Randomization tests showed patterns distinct from real data only for the young duplicates (supplementary fig. S7*A* and *B*, Supplementary Material online), indicating a biological pattern rather than a data bias. Thus PIC on the trees after diagnostic plot tests supports the ortholog conjecture for young duplicates, whereas the inference remains biased for older duplicates.

### Approach 2: PIC with Branch Length Transformation

Most phylogenetic methods are developed for the Brownian model of trait evolution, including PIC (Felsenstein 1985; Cornwell and Nakagawa 2017). Deviations from pure BM violate the fundamental assumptions of PIC applicability and can affect its performance for testing hypotheses about correlated evolution (Garland 1992; Garland et al. 1992; Díaz-Uriarte and Garland 1996, 1998). Using model fitting (see Materials and Methods), we found that 75.6% gene trees (supplementary fig. S8, Supplementary Material online) supported the OU model. Remedial measures, such as branch length transformations and diagnostic tests, can substantially recover the performance of the PIC methods when character evolution is not BM, or when contrasts are nonindependent of the phylogeny (Garland et al. 1992; Díaz-Uriarte and Garland 1996, 1998).

**Fig. 4.**
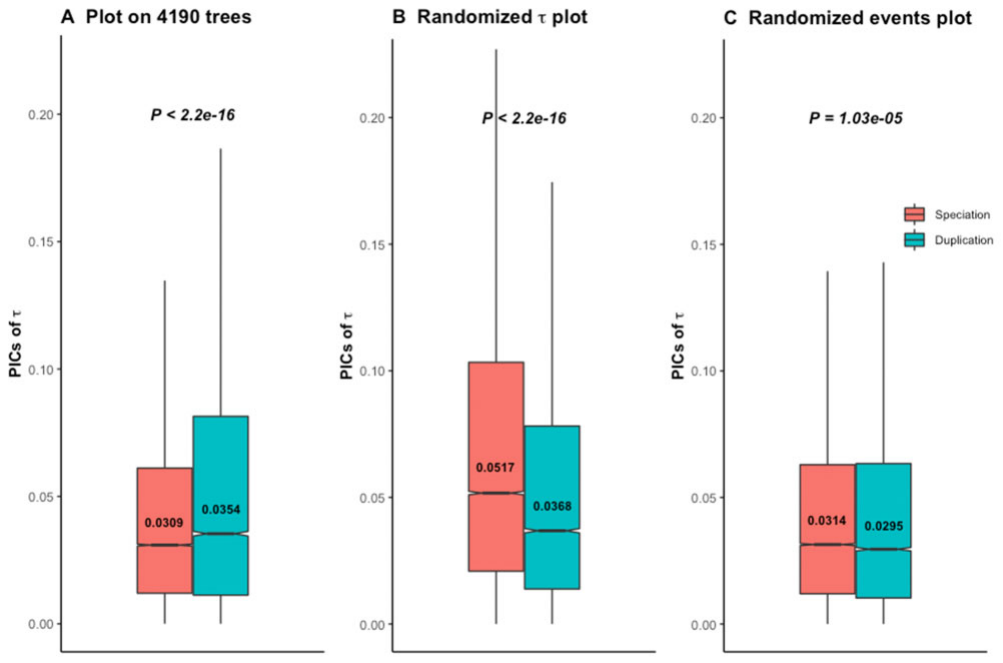
The ortholog conjecture test for contrasts standardized branch transformed trees. *P* values are from Wilcoxon two-tailed tests. Values inside boxplots denote median PIC value of the corresponding event. (*A*) Using 4,190 out of 4,288 calibrated trees that passed diagnostic tests following branch length transformation. (*B*) Permuting τ and (*C*) permuting internal events on contrasts standardized branch length transformed trees produce distinct patterns compared with the empirical gene trees of (*A*).

We thus applied branch length transformation on all 4,288 trees, along with diagnostic tests for consistency. The 4,190 trees (97.7%) which pass diagnostic tests after branch length transformation support the ortholog conjecture (fig. 4*A*). Due to the lack of absolute age for these transformed trees, we did not distinguish young and old duplicates. Applying such branch length transformation then diagnostic tests to the gene trees of Dunn et al., we also found support for the ortholog conjecture in 98.8% (8,417 out of 8,520) (supplementary fig. S9*A*, Supplementary Material online), as well as for 99.9% (2,080 out of 2,082) of their trees with strong phylogenetic signal (supplementary fig. S10*A*, Supplementary Material online). Randomization tests on all these sets of trees following branch length transformations clearly showed distinct patterns compared with the empirical data (fig. 4*B* and *C*; supplementary figs. S9*B*, S9*C*, S10*B*, and S10*C*, Supplementary Material online), indicating that results are not due to inference bias once the data are properly transformed.

### Approach 3: Phylogenetic Data Modeling

State-dependent model fitting allows to compare the evolutionary rates (σ^2^) and the changes in adaptive optimum value (θ) associated with specific states (speciation or duplication) for each tree (Beaulieu et al. 2012; Clavel et al. 2015). Under the ortholog conjecture, our expectation is that there should be more shifts in optimum value of τ between paralogs than between orthologs. Moreover, the evolutionary rates after duplication should be higher than after speciation (σ^2^_duplication_ > σ^2^_speciation_). Of course, trends on empirical data should differ from randomized ones. When we modeled the evolution of τ (see Materials and Methods), 32 out of 4,288 trees failed to fit any model due to invariance in τ. Among the others, 308 supported BM1, 704 BMM, 2,874 OU1, and 370 OUM, as the best-fit models (supplementary fig. S8, Supplementary Material online). We performed our analyses separately for young and old duplicates.

On the 8.6% multi optima trees (OUM) the optimum value are significantly higher for duplications, both young and old (θ_dup_ > θ_spe_) (supplementary table S2, Supplementary Material online). Thus, paralogs shift toward higher tissue specificity. These results are not observed on randomized trees, supporting a biological pattern in the data (supplementary table S2, Supplementary Material online).

We also applied a Bayesian method (Uyeda and Harmon 2014) to quantify the number of adaptive optimum shifts, as suggested for small trees (Cooper, Thomas, Venditti, et al. 2016). Unlike the other approach, such detection of evolutionary shifts in a phylogeny does not need a priori knowledge of different states on the tree. Using a strict posterior probability threshold of ≥0.7 with this method, we find that most optimum shifts per branch for τ follow duplications (median after speciation: 0%, after duplication: 12.5%, paired two-sided Wilcoxon rank-sum test *P *<* *2.2e^−16^). An OU model can often be incorrectly favored over a BM model in a maximum likelihood framework when applied to trees with <200 tips (Cooper, Thomas, Venditti, et al. 2016). Our gene trees have a median of only 15 tips. We thus applied a conservative Bayesian approach on all of the 3,244 trees for which OU was the preferred model (OU1 + OUM). Even with such a strict posterior probability threshold of ≥0.7, 1,101 trees (33.9%) still supported the OUM model, including 901 trees identified as OU1 by maximum likelihood. We detected the same trend of optimum shifts per branch (median after speciation: 2.3%, after duplication: 10%, paired Wilcoxon rank-sum test *P *<* *2.2e^−16^). These results are largely consistent for both young and old duplicates (table 2; supplementary table S3, Supplementary Material online). However, the rates of optimum shifts are faster only for young duplicates (table 2; supplementary table S3, Supplementary Material online).

**Table 2.** Summary Statistics on 1,101 OUM Trees Passing a Posterior Probability Cutoff of ≥0.7 in a Bayesian Framework.

Duplication Age	Proportions of Regime Shifts per Branch		Paired Two-Sided Wilcoxon Rank Sum Test	Regime Shift Rates (shifts/My)		Two-Sided Wilcoxon Rank Test
	After Speciation	After Duplication		After Speciation	After Duplication	
Young	3.1%	4.5%	3.4e^−12^	0.013	0.031	1.7e^−11^
Old	2.6%	10%	<2.2e^−16^	0.013	0.0023	<2.2e^−16^

Note.—Above analyses include 13,824 speciation, 3,027 young, and 2,814 old duplication events. Values shown in the table indicate median values. The difference in proportions of regime shifts per branch after speciation events for two types of duplications is due to the different sets of trees used. Few trees shared both types of duplicates. Proportion of regime shifts per branch of events is estimated for each tree, and thus paired Wilcoxon test is used to compare the difference. A single gene tree can have multiple-optima shift rates for events, and thus two-sided Wilcoxon rank test was used for comparison.

Analyses on the trees where σ^2^ varies between events (BMM) also support the ortholog conjecture for young duplicates (table 3). Randomized data showed distinct patterns from empirical data. However, again there was neither support for the ortholog conjecture nor signal relative to randomization for the old duplicates.

**Table 3.** Summary Statistics for Brownian Trees.

Duplication Age	Data	σ^2^_Speciation_	σ^2^_Duplication_	σ^2^_Duplication/_σ^2^_Speciation_	*P*-value
Young	Empirical (*n*_Speciation_= 4,642; *n*_Duplication_ = 1,742)	9e^−5^	1.4e^−4^	1.5	5e^−12^
	Randomized τ (*n*_Speciation_= 4,618; *n*_Duplication_ = 1,723)	6.9e^−4^	2.2e^−4^	0.32	1.4e^−13^
	Randomized events (*n*_Speciation_= 3,215; *n*_Duplication_ = 1,438)	1.7e^−4^	8.5e^−5^	0.5	0.02
Old	Empirical (*n*_Speciation_= 5,356; *n*_Duplication_ = 1,295)	1.7e^−4^	2e^−9^	1.2e^−5^	<2.2e^−16^
	Randomized τ (*n*_Speciation_= 5,337; *n*_Duplication_ = 1,291)	9.1e^−4^	2.5e^−10^	2.7e^−7^	<2.2e^−16^
	Randomized events (*n*_Speciation_= 2,788; *n*_Duplication_ = 800)	1.8e^−4^	2.1e^−9^	1.2e^−5^	<2.2e^−16^

Note.—Median values of σ^2^ are shown; *P*-value from paired two-sided Wilcoxon test.

## Discussion

We agree with Dunn et al. (2018) that evolutionary comparisons should be done considering a phylogenetic framework when possible. However, this does not imply that phylogenetic methods can be applied easily to phylogenomics. To get a clear picture, we limited our study to the same gene trees used by Dunn et al. (2018). Our reanalysis identified problems generated by the time calibration of old duplication nodes of pruned trees, the inclusion of pure speciation gene trees, and violations of the Brownian model. The strongest bias was for duplication nodes preceding the oldest speciation nodes. This, in turn, introduced several biases in the analyses and influenced results.

When we identified and controlled for such biases, PIC results changed to support the ortholog conjecture, consistent with our previous pairwise analysis (Kryuchkova-Mostacci and Robinson-Rechavi 2016) on the same τ data. Our fundamental point is that the conclusions drawn by Dunn et al., but also by anyone else who will have followed the same approach of applying PIC to gene trees, are not reliable unless extreme care is taken. This is because gene trees with orthologs and paralogs have more complex evolutionary histories, and different sampling biases, than species trees for which these methods were developed.

To date, a few studies have applied phylogenetic comparative methods to understand the effect of gene duplication on functional evolution (Oakley et al. 2005, 2006; Eng et al. 2009; Rohlfs and Nielsen 2015; Dunn et al. 2018; Fukushima and Pollock 2020). None before Dunn et al. applied PIC method to compare speciation and duplication events on the same trees using a single continuous trait. Such application requires thorough testing of the fundamental assumptions of the method on such time-calibrated trees (Garland 1992; Garland et al. 1992; Díaz-Uriarte and Garland 1996, 1998; Freckleton 2000; Freckleton and Harvey 2006; Cooper, Thomas, FitzJohn, et al. 2016). Hence, we explored whether the application of a phylogenetic method might inflate errors if applied without assumption testing, typically by rejecting of the null hypothesis under the null (simulations or randomizations). Indeed, it is the case (fig. 1*A* and *B*). Along with the calibration bias for old duplication nodes, the relative ages of the speciation and duplication events strongly differ in these trees due to the choice of species. Using such trees without control for biases may bring about lack of statistical power to detect the signal of ortholog conjecture, and even bias toward an opposite pseudosignal.

Time calibration of ancient duplication events is one of the major issues we uncovered. The approach of Dunn et al. considered pruned trees with available trait (τ here) data for time calibration using speciation time points (see Materials and Methods). Such pruned trees often have many duplication nodes older than the oldest speciation nodes. Sequence-based evolutionary rate (e.g., d*N*/d*S*) analyses in different species have found higher sequence evolutionary rate following gene duplication (Conant and Wagner 2003; Kim and Yi 2006; Scannell and Wolfe 2007; Han et al. 2009; Studer and Robinson-Rechavi 2009; Panchin et al. 2010; Pegueroles et al. 2013; Pich i Roselló and Kondrashov 2014; Holland et al. 2017). Therefore, calibration bias is not surprising for those duplication nodes in the absence of time constraints (supplementary fig. S4*A*–*C* and table S1, Supplementary Material online). Instead, we performed time calibration before pruning, so that the oldest speciation time points can provide upper age limits and reduce calibration bias (supplementary fig. S4*D*–*F*, Supplementary Material online). This is strongly recommended because the performance of the phylogenetic methods relies on accurate branch length information, especially for multistate univariate trait analysis.

Dunn et al. (2018) performed several analyses (e.g., added random noise in the speciation calibration time points, extended terminal branch length, removed old duplication nodes) to take into account issues with branch lengths, but their simulations and our randomization tests show that they were insufficient to correct for this bias (fig. 2*A* and *C*). Dunn et al. also provided the hutan::picx() R function to compute PIC for OU trees. In their simulation-based function, they estimated ancestral states by the “GLS_OUS” method using the bias calibrated phylogeny. Therefore, their method does not add anything specific to deal with the OU trees. As they did not control for phylogenetic independence of the contrasts, and did not consider the relative ages of the speciation and old duplication events, they always obtained lower PIC of duplication events. Due to such phylogenetic internal parameter dependence, their PIC analyses produced similar trends with real or randomized data.

Assumptions of proper branch length information and of BM of trait evolution are related, so that modifications of branch lengths can change the evolutionary model (Díaz-Uriarte and Garland 1996, 1998). Contrasting a single rate OU to BM models, Dunn et al. (2018) identified 99.9% gene trees which favored an OU model, more explicitly an OU1 model. This appears to be 67% when we performed multivariate data modeling in a maximum likelihood framework on trees with less or no calibration bias (supplementary fig. S8, Supplementary Material online). PIC analyses with diagnostic tests provided weak support for the ortholog conjecture for the young duplicates (fig. 3*A*–*C*), in contrast to previous results of Dunn et al. A small effect size in our inference is not surprising as PIC is applied on OU trees. Similar patterns of results from empirical and randomization tests for the old duplicates indicate that one should be extremely careful before integrating them into a phylogenetic analysis. Branch length transformation attempts to transform the OU trees to BM trees to meet the underlying assumption of phylogenetic comparative method (Butler and King 2004). Hence, it can address the issue of low power when underlying assumptions of phylogenetic methods are violated (Díaz-Uriarte and Garland 1996, 1998). Following this approach along with the diagnostic tests, we obtained substantial support for the ortholog conjecture (fig. 4*A*–*C*; supplementary figs. S9 and S10, Supplementary Material online).

Phylogenetic data modeling also appears to be a powerful tool for such hypothesis testing, where one can estimate the trait evolutionary rates or optima shift rates per event without transforming OU trees to BM trees. More support for the OU trees (supplementary fig. S8, Supplementary Material online) could be due to the fact that we performed multivariate evolutionary model fitting mostly on small trees (Cooper, Thomas, Venditti, et al. 2016). Among them only 8.6% trees supported the OUM model. Following the recommendation of Cooper, Thomas, Venditti, et al. (2016), we applied a Bayesian approach on small trees to identify multi optima trees. Although previous studies (Uyeda and Harmon 2014; Khabbazian et al. 2016; Uyeda et al. 2017) have suggested a liberal cutoff of ≥0.2 to detect an optimum shift with a Bayesian approach, we used a strict posterior probability cutoff of ≥0.7. We performed our analyses on the 33.9% of OUM trees passing such a strict posterior probability threshold. Our results from the PIC analyses with controls were also supported by the maximum likelihood and Bayesian data modeling approaches. This shows that once proper precautions are taken, the empirical trends do not depend on the number of selected gene trees or of internal node events included.

Empirical support for the ortholog conjecture has been mixed, with some studies supporting it (Koonin 2005; Studer and Robinson-Rechavi 2009; Altenhoff et al. 2012; Chen and Zhang 2012; Gabaldón and Koonin 2013; Rogozin et al. 2014; Kryuchkova-Mostacci and Robinson-Rechavi 2016; Fukushima and Pollock 2020), and a few failing to do so (Nehrt et al. 2011; Dunn et al. 2018; Stamboulian et al. 2020). Our results provide additional support for the ortholog conjecture using tissue specificity data in a phylogenetic framework after controlling for biases. Due to lack of detailed functional information, many studies are still limited to gene expression data as a proxy of function. Recently, using a functional replaceability assay, experimental studies (Kachroo et al. 2015; Laurent et al. 2020) have shown that orthologous genes can be swapped between essential yeast genes and human, although this is rarely the case for all the members of expanded human gene families (Laurent et al. 2020), validating one prediction of the ortholog conjecture.

## Materials and Methods

### Data Reproducibility Details

Our analyses are based on 21,124 gene trees obtained from ENSEMBL Compara v.75 (Herrero et al. 2016) as used by Dunn et al. (2018). We used the same random seed number as in Dunn et al. (2018) to reproduce the simulation results for reanalysis. All reproduced data of Dunn et al. were stored in the “manuscript_dunn.RData” file (https://doi.org/10.5281/zenodo.4003391). We used the results stored in the “data” or “phylo” slot of the trees for further analyses. To differentiate our own function from theirs (Dunn et al. 2018), we renamed the original function script of Dunn et al. from “functions.R” to “functions_Dunn.R.” We made separate scripts for PIC analyses (Premanuscript_run_TMRR.R) and data modeling analyses (Model_fitting.R). Some of the analyses were time consuming, so we stored our outputs in “Analyses_TMRR.RData” and “Model_fitting_TMRR.Rdata” files (https://doi.org/10.5281/zenodo.4003391) to load during analyses. All the details of different functions are provided inside the scripts. We supply all the previously stored data (to reduce computation time during reproduction of result) and function files including our own (functions_TM_new.R) with this study. All scripts are available on GitHub: https://github.com/tbegum/Testing_the_ortholog_conjecture.

### Fixing Time Calibration Bias of Duplication Nodes

We first present the approach that Dunn et al. (2018) used, for clarity. When two speciation nodes had the same label in the gene tree, Dunn et al. edited the more recent one to “NA” rather than “speciation.” Indeed the presence of the same clade names at different node depths forces all the intervening branches to have length zero when the tree is time calibrated, leading to failure of calibration (Dunn et al. 2018). For trait evolution, they annotated the tips of these modified trees with precomputed tissue specificity data, τ from eight vertebrate species (human, gorilla, chimpanzee, macaque, mouse, opossum, platypus, and chicken) (from Kryuchkova-Mostacci and Robinson-Rechavi [2016]). τ is a univariate index between 0 and 1 that measures tissue specificity of gene expression (Yanai et al. 2005): τ close to 1 indicates high tissue specificity, whereas close to 0 indicates more ubiquitous expression. Here, τ was computed across six tissues: brain, cerebellum, heart, kidney, liver, and testis, based on the RNA-seq data of Brawand et al. (2011). Dunn et al. pruned the gene trees to remove tips with missing τ data and then time calibrated them using speciation clade ages in the chronos() function with the “correlated” model from the R package “ape” (Paradis et al. 2004). The modified NA clades were not used for this calibration. They used seven speciation time points with a maximum age of 296 My. Thus, they obtained 8,520 calibrated gene trees having at least four tips with nonnull trait data (table 1; supplementary fig. S4*A*–*C*, Supplementary Material online). Among these trees, 2,990 were pure speciation trees, which include 12,919 speciation events, or 19% of all speciation nodes.

Relative to Dunn et al., we exchanged the order of pruning and time calibration steps, that is, we first time calibrated the 21,124 modified (i.e., with NA added) gene trees, followed by pruning to have at least four tips with τ data. This makes use of all 32 available speciations time points and helps to limit the calibration bias of the old duplication events (supplementary fig. S4*D*–*F*, Supplementary Material online). Calibration fails for some trees, and we obtained 7,336 calibrated gene trees. The maximum node age of old duplication events is 1,175.2 My for these trees, as opposed to 11,799,977 My (older than the universe) for the trees obtained by the original approach (table 1; supplementary table S1, Supplementary Material online). Among these 7,336 gene trees, we kept 4,288 which have at least one speciation and one duplication events; we removed 39 pure duplication and 3,009 pure speciation trees. This 4,288 gene tree set is our basis for evaluating phylogenetic methods’ capacity to test the ortholog conjecture (table 1): We compare the evolutionary rates, σ^2^, or PICs of speciation and duplication events of the same genes.

### Model Selection for τ Evolution

We followed a state-dependent model-fitting approach to identify BM or OU trees. We classified time-calibrated gene duplication nodes as “young” (≤296 My, the maximum speciation age) or “old” (>296 My) before model fitting. We performed stochastic mapping of our gene trees by assigning discrete states (“speciation,” “young-duplication,” “old-duplication,” and “NA”) to the branches based on the corresponding ancestral node events using the simmap() function of the phytools R package (Revell 2012). For each mapped tree, we fitted four different models of τ evolution using maximum likelihood: 1) BM1, a single BM rate of evolution (i.e., σ^2^_speciation_ = σ^2^_young-duplication_ = σ^2^_old-duplication_); 2) BMM, a BM with multiple rates of evolution for different events (i.e., different σ^2^ are allowed); 3) OU1, a single optimum OU model (i.e., θ_speciation_ = θ_young-duplication_ = θ_old-duplication,_ σ^2^_speciation_ = σ^2^_young-duplication_ = σ^2^_old-duplication,_ α _speciation_ = α_young-duplication_ = α _old-duplication_), and 4) OUM, a multi optimum OU model with identical strength of selection and rate of drift acting on all selective regimes (i.e., like OU1 but θ_speciation_ ≠ θ_young-duplication_ ≠ θ_old-duplication_).

We used both the mvMORPH (Clavel et al. 2015) and OUwie (Beaulieu et al. 2012) R packages to perform model fitting. Sometimes the information contained within a tree is insufficient with respect to the complexity of the fitted models. This can lead to poor model choice by returning a log-likelihood that is suboptimal and may provide incorrect estimation of one or more model parameters for that tree (Beaulieu et al. 2012). Hence, we included the diagnostics (diagnostic=T or diagn=T) during model fitting. The Eigen values of the Hessian matrix of the diagnostics indicate whether convergence of the model has been achieved or whether the parameter estimates are reliable (Beaulieu et al. 2012). For the BM1, BMM, OU1, and OUM models, we first fitted the model using mvMORPH for each gene tree. If any of the models failed to converge for the tree or if the Eigen values of the Hessian matrix indicated that it was not reliable, we refitted that model using OUwie to include it in model comparison. If still it failed, we removed that model for that tree. For model comparisons on each gene tree, we calculated the Akaike weights (ω) for each fitted model by means of the second-order Akaike information criteria (AICc), which includes a correction for small sample sizes (Akaike 1974; Burnham and Anderson 2002). The model with highest ω was selected as the best-supported model of τ evolution for the tree (Burnham and Anderson 2002; Gearty et al. 2018). We estimated model parameters for each tree based on the best-fit model.

### Bayesian Modeling to Detect Phenotypic Optimum Shift

Regime shifts, that is, shifts of optimal τ values, in OU models were detected by a Bayesian phylogenetic approach of the bayou R package (Uyeda and Harmon 2014). The reversible-jump phylogenetic comparative approach was used to perform MCMC sampling of locations, magnitudes and numbers of shifts in multiple-optima OU models. We ran MCMC chains for 100,000 generations, and the first 30% of samples were dropped as burn-in. We used a strict threshold of posterior probability ≥0.7 to detect an adaptive shift at a given branch of the phylogeny. For each event (“speciation” or “duplication”), we used a ratio of the number of optimum shifts to the number of branches for that event to estimate the proportions of shifts in a phylogeny.

### Randomization Test of τ Values

For each tree, we used τ data (column name “Tau” in each tree “data” object) across the tips to carry out our randomization test. To randomize, we permuted the actual τ data without altering internal node events. The pic() function of the “ape” package (Paradis et al. 2004) was used to compute PIC of nodes for each tree using permuted τ of tips. For each run, we compared the contrasts of speciation and duplication events of the whole set of randomized trees to estimate difference in event contrasts based on Wilcoxon signed rank test. For 100 runs, we repeated the above process 100 times to obtain a distribution plot of 100 independent *P* values. For our model-fitting approach, we used the same empirical simmap trees with permuted τ data at the tips. We reestimated the model parameters of the randomized τ trees using the best-fit model chosen for the corresponding empirical gene trees.

### Randomization Test of Node Events

Some of the speciation nodes had daughters with same clade names in the gene trees we used for our study. Dunn et al. changed such node events to “NA” to avoid problems during time calibration of the trees. Such annotated node event information (“Speciation,” “Duplication,” “NA”) for each tree was available as “Event” in the tree “data” slot. To randomize, we permuted the internal node events (added as column name “event_new” in the “data” slot) by maintaining the actual proportion of events for each tree. Then, we used the PIC of actual τ at tips to estimate contrasts difference between newly assigned speciation and duplication node events by Wilcoxon rank tests. For 100 independent runs, we repeated the same procedure to obtain 100 independent *P* values. As the internal node events were changed after such randomization, we reclassified gene duplication nodes as “young” or “old” on the event modified trees and repainted the trees. We reestimated the model parameters for the discrete states of the randomized event trees using the best-fit model chosen for the corresponding empirical gene trees.

### Checking for Contrasts Standardization by Diagnostic Tests

We used several additional diagnostic tests on those trees to identify adequate independent nodes contrast standardization before drawing any inference by PIC method, as recommended in several studies (Garland 1992; Díaz-Uriarte and Garland 1996, 1998; Freckleton and Harvey 2006; Cooper, Thomas, FitzJohn, et al. 2016). The most usual method for contrasts standardization is to check a correlation between the absolute values of PICs and their expected standard deviations (i.e., square root of sum of branch lengths) (Garland et al. 1992; Díaz-Uriarte and Garland 1998; Cooper, Thomas, FitzJohn, et al. 2016). Under BM, there should be no correlation. This correlation test and another between the absolute values of PICs and the logarithm of their node age are model diagnostic tests in the caper (Comparative Analyses of Phylogenetics and Evolution in R) package (Purvis and Rambaut 1995; Cooper, Thomas, FitzJohn, et al. 2016; Orme 2018; R Core Team 2018). We used both of them by using the “crunch” algorithm of the caper package, which implements the methods originally provided in CAIC (Purvis and Rambaut 1995; Cooper, Thomas, FitzJohn, et al. 2016; Orme 2018; R Core Team 2018). Correlation of node heights with absolute values of contrasts or PICs has also been reported to be a reliable indicator of deviation from the Brownian model (Freckleton and Harvey 2006). Hence, we computed node height for each node in a tree using the ape package (Paradis et al. 2004). We also used the correlations of node height and node depth to the absolute value of nodes contrasts to rule out significant trend in any of the four tests. We used *P *<* *0.05 to assess a significant correlation for the diagnostic tests. A significant trend (positive or negative) indicates phylogenetic dependence for that tree (Garland 1992; Garland et al. 1992; Díaz-Uriarte and Garland 1998; Freckleton and Harvey 2006; Cooper, Thomas, FitzJohn, et al. 2016), and we removed those trees from our analysis. Contrast calculation on negative branch lengths is not desirable, so we removed trees with negative branch lengths before applying the crunch() function. To assure that nodes contrast standardization is independent of the phylogeny, we considered sets of trees passing all four diagnostic tests for further analyses.

### Branch Length Transformation

Transformation of branch lengths has been proposed to restore the performance of PIC method when the true evolutionary model is not BM or is unknown, or when branch lengths are in error (Garland et al. 1992; Díaz-Uriarte and Garland 1996, 1998). In such cases, branch lengths are transformed by raising a family power of branch length ranging from 0 to 2 in intervals of 0.1, plus the log_10_ of the branch lengths (Díaz-Uriarte and Garland 1996, 1998). For each transformation, the program computes the correlation between the absolute value of the standardized contrasts and their standard deviations until no significant correlation is obtained, to ensure adequate independent contrasts standardization (Díaz-Uriarte and Garland 1996, 1998). Finally, we excluded trees for which adequate contrasts standardization is not achieved even after raising the branch length power to 2 (Díaz-Uriarte and Garland 1996, 1998).

### Details of Other Packages Used in This Study

We used phylosig function() of the phytools package (Revell 2012) to identify trees with phylogenetic signal (*P *<* *0.05) using Blomberg’s *K* (Blomberg et al. 2003; Münkemüller et al. 2012; Revell 2012). Analyses and plotting were performed in R version 3.5.1 (R Core Team 2018) using treeio (Guangchuang 2018), ggtree (Guangchuang et al. 2017), stringr (Wickham 2019), digest (Antoine Lucas et al. 2018), dplyr (Wickham et al. 2017), tidyverse (Wickham 2017), ggrepel (Slowikowski 2018), gtools (Warnes et al. 2018), ggplot2 (Wickham 2016), cowplot (Wilke 2019), easyGgplot2 (Kassambara 2014), gridExtra (Auguie 2017), and png (Urbanek 2013) libraries. 

## Supplementary Material

Supplementary data are available at *Molecular Biology and Evolution* online.

## Supplementary Material

msaa288_Supplementary_Data

## References

[msaa288-B1] Akaike H. 1974. New look at statistical-model identification. IEEE Trans Automat Contr. 19(6):716–723.

[msaa288-B2] Altenhoff AM , Studer RA, Robinson-Rechavi M, Dessimoz C. 2012. Resolving the ortholog conjecture: orthologs tend to be weakly, but significantly, more similar in function than paralogs. PLoS Comput Biol. 8(5):e1002514. 22615551 10.1371/journal.pcbi.1002514 PMC3355068

[msaa288-B3] Antoine Lucas DE , Tuszynski J, Bengtsson H, Urbanek S, Frasca M, Lewis B, Stokely M, Muehleisen H, Murdoch D, Hester J, et al 2018. Digest: Create compact hash digests of r objects. Available from: https://CRAN.R-project.org/package=digest

[msaa288-B4] Auguie B. 2017. GridExtra: miscellaneous functions for “grid” graphics. Available from: https://CRAN.R-project.org/package=gridExtra

[msaa288-B5] Beaulieu JM , Jhwueng DC, Boettiger C, O’Meara BC. 2012. Modeling stabilizing selection: expanding the Ornstein-Uhlenbeck model of adaptive evolution. Evolution 66(8):2369–2383. 22834738 10.1111/j.1558-5646.2012.01619.x

[msaa288-B6] Benjamini Y , Yekutieli D. 2005. Quantitative trait loci analysis using the false discovery rate. Genetics 171(2):783–790. 15956674 10.1534/genetics.104.036699 PMC1456787

[msaa288-B7] Blomberg SP , Garland T, Ives AR. 2003. Testing for phylogenetic signal in comparative data: behavioral traits are more labile. Evolution 57(4):717–745. 12778543 10.1111/j.0014-3820.2003.tb00285.x

[msaa288-B8] Brawand D , Soumillon M, Necsulea A, Julien P, Csárdi G, Harrigan P, Weier M, Liechti A, Aximu-Petri A, Kircher M, et al 2011. The evolution of gene expression levels in mammalian organs. Nature 478(7369):343–348. 22012392 10.1038/nature10532

[msaa288-B9] Burnham K , Anderson D. 2002. Model selection and multimodel inference. New York: Springer.

[msaa288-B10] Butler MA , King AA. 2004. Phylogenetic comparative analysis: a modeling approach for adaptive evolution. Am Nat. 164(6):683–695. 29641928 10.1086/426002

[msaa288-B11] Catalán A , Briscoe AD, Höhna S. 2019. Drift and directional selection are the evolutionary forces driving gene expression divergence in eye and brain tissue of *Heliconius butterflies*. Genetics 213(2):581–594. 31467133 10.1534/genetics.119.302493 PMC6781903

[msaa288-B12] Chen J , Swofford R, Johnson J, Cummings BB, Rogel N, Lindblad-Toh K, Haerty W, Palma FD, Regev A. 2019. A quantitative framework for characterizing the evolutionary history of mammalian gene expression. Genome Res. 29(1):53–63. 30552105 10.1101/gr.237636.118 PMC6314168

[msaa288-B13] Chen X , Zhang J. 2012. The ortholog conjecture is untestable by the current gene ontology but is supported by RNA sequencing data. PLoS Comput Biol. 8(11):e1002784. 23209392 10.1371/journal.pcbi.1002784 PMC3510086

[msaa288-B14] Clavel J , Escarguel G, Merceron G. 2015. mvMORPH: an R package for fitting multivariate evolutionary models to morphometric data. Methods Ecol Evol. 6(11):1311–1319.

[msaa288-B15] Conant GC , Wagner A. 2003. Asymmetric sequence divergence of duplicate genes. Genome Res. 13(9):2052–2058. 12952876 10.1101/gr.1252603 PMC403682

[msaa288-B16] Cooper N , Thomas GH, FitzJohn RG. 2016. Shedding light on the ‘dark side’ of phylogenetic comparative methods. Methods Ecol Evol. 7(6):693–699. 27499839 10.1111/2041-210X.12533 PMC4957270

[msaa288-B17] Cooper N , Thomas GH, Venditti C, Meade A, Freckleton RP. 2016. A cautionary note on the use of Ornstein Uhlenbeck models in macroevolutionary studies. Biol J Linn Soc Lond. 118(1):64–77. 27478249 10.1111/bij.12701 PMC4949538

[msaa288-B18] Cornwell W , Nakagawa S. 2017. Phylogenetic comparative methods. Curr Biol. 27(9):R333–R336. 28486113 10.1016/j.cub.2017.03.049

[msaa288-B19] Díaz-Uriarte R , Garland T. 1996. Testing hypotheses of correlated evolution using phylogenetically independent contrasts: sensitivity to deviations from Brownian motion. Syst Biol. 45(1):27–47.

[msaa288-B20] Díaz-Uriarte R , Garland T. 1998. Effects of branch length errors on the performance of phylogenetically independent contrasts. Syst Biol. 47(4):654–672. 12066309 10.1080/106351598260653

[msaa288-B21] Dunn CW , Zapata F, Munro C, Siebert S, Hejnol A. 2018. Pairwise comparisons across species are problematic when analyzing functional genomic data. Proc Natl Acad Sci U S A. 115(3):E409–E417. 29301966 10.1073/pnas.1707515115 PMC5776959

[msaa288-B22] Eastman JM , Alfaro ME, Joyce P, Hipp AL, Harmon LJ. 2011. A novel comparative method for identifying shifts in the rate of character evolution on trees. Evolution 65(12):3578–3589. 22133227 10.1111/j.1558-5646.2011.01401.x

[msaa288-B23] Eng KH , Bravo HC, Keleş S. 2009. A phylogenetic mixture model for the evolution of gene expression. Mol Biol Evol. 26(10):2363–2372. 19602540 10.1093/molbev/msp149 PMC2738779

[msaa288-B24] Felsenstein J. 1985. Phylogenies and the comparative method. Am Nat. 125(1):1–15.

[msaa288-B25] Freckleton R. 2000. Phylogenetic tests of ecological and evolutionary hypotheses: checking for phylogenetic independence. Funct Ecol. 14(1):129–134.

[msaa288-B26] Freckleton R , Harvey P, Pagel M. 2002. Phylogenetic analysis and comparative data: a test and review of evidence. Am Nat. 160(6):712–726. 18707460 10.1086/343873

[msaa288-B27] Freckleton RP , Harvey PH. 2006. Detecting non-Brownian trait evolution in adaptive radiations. PLoS Biol. 4(11):e373. 17090217 10.1371/journal.pbio.0040373 PMC1634878

[msaa288-B28] Fukushima K , Pollock DD. 2020. Amalgamated cross-species transcriptomes reveal organ-specific propensity in gene expression evolution. *Nat Commun.* 11:4459. 10.1038/s41467-020-18090-8 PMC7479108 32900997

[msaa288-B29] Gabaldón T , Koonin EV. 2013. Functional and evolutionary implications of gene orthology. Nat Rev Genet. 14(5):360–366. 23552219 10.1038/nrg3456 PMC5877793

[msaa288-B30] Garland T. 1992. Rate tests for phenotypic evolution using phylogenetically independent contrasts. Am Nat. 140(3):509–519. 19426053 10.1086/285424

[msaa288-B31] Garland TJ , Harvey P, Ives A. 1992. Procedure for the analysis of comparative data using phylogenetically independent contrasts. Syst Biol. 41(1):18–32.

[msaa288-B32] Gearty W , McClain CR, Payne JL. 2018. Energetic tradeoffs control the size distribution of aquatic mammals. Proc Natl Acad Sci U S A. 115(16):4194–4199. 29581289 10.1073/pnas.1712629115 PMC5910812

[msaa288-B33] Grafen A. 1989. The phylogenetic regression. Philos Trans R Soc Lond B Biol Sci. 326(1233):119–157. 2575770 10.1098/rstb.1989.0106

[msaa288-B34] Guangchuang Y. 2018. Treeio: Base classes and functions for phylogenetic tree input and output. Available from: https://guangchuangyu.github.io/software/treeio.

[msaa288-B35] Guangchuang Y , David S, Huachen Z, Yi G, Tommy T-YL. 2017. Ggtree: an R package for visualization and annotation of phylogenetic trees with their covariates and other associated data. Methods Ecol Evol. 8(1):28–36.

[msaa288-B36] Han MV , Demuth JP, McGrath CL, Casola C, Hahn MW. 2009. Adaptive evolution of young gene duplicates in mammals. Genome Res. 19(5):859–867. 19411603 10.1101/gr.085951.108 PMC2675974

[msaa288-B37] Hansen TF. 1997. Stabilizing selection and the comparative analysis of adaptation. Evolution 51(5):1341–1351. 28568616 10.1111/j.1558-5646.1997.tb01457.x

[msaa288-B38] Herrero J , Muffato M, Beal K, Fitzgerald S, Gordon L, Pignatelli M, Vilella AJ, Searle SM, Amode R, Brent S, et al 2016. Ensembl comparative genomics resources. Database 2016:baw053. 10.1093/database/baw053 PMC4852398 27141089

[msaa288-B39] Hochberg Y , Benjamini Y. 1990. More powerful procedures for multiple significance testing. Stat Med. 9(7):811–818. 2218183 10.1002/sim.4780090710

[msaa288-B40] Holland PW , Marlétaz F, Maeso I, Dunwell TL, Paps J. 2017. New genes from old: asymmetric divergence of gene duplicates and the evolution of development. Philos Trans R Soc Lond B Biol Sci. 372:20150480. 27994121 10.1098/rstb.2015.0480 PMC5182412

[msaa288-B41] Kachroo AH , Laurent JM, Yellman CM, Meyer AG, Wilke CO, Marcotte EM. 2015. Evolution. systematic humanization of yeast genes reveals conserved functions and genetic modularity. Science 348(6237):921–925. 25999509 10.1126/science.aaa0769 PMC4718922

[msaa288-B42] Kassambara A. 2014. EasyGgplot2: perform and customize easily a plot with ggplot2. Available from: htt://www.sthda.com.

[msaa288-B43] Khabbazian M , Kriebel R, Rohe K, Ané C. 2016. Fast and accurate detection of evolutionary shifts in ornstein-uhlenbeck models. Methods Ecol Evol. 7(7):811–824.

[msaa288-B44] Kim SH , Yi SV. 2006. Correlated asymmetry of sequence and functional divergence between duplicate proteins of saccharomyces cerevisiae. Mol Biol Evol. 23(5):1068–1075. 16510556 10.1093/molbev/msj115

[msaa288-B45] Koonin EV. 2005. Orthologs, paralogs, and evolutionary genomics. Annu Rev Genet. 39(1):309–338. 16285863 10.1146/annurev.genet.39.073003.114725

[msaa288-B46] Kryuchkova-Mostacci N , Robinson-Rechavi M. 2016. Tissue-specificity of gene expression diverges slowly between orthologs, and rapidly between paralogs. PLoS Comput Biol. 12(12):e1005274. 28030541 10.1371/journal.pcbi.1005274 PMC5193323

[msaa288-B47] Laurent JM , Garge RK, Teufel AI, Wilke CO, Kachroo AH, Marcotte EM. 2020. Humanization of yeast genes with multiple human orthologs reveals functional divergence between paralogs. PLoS Biol. 18(5):e3000627. 32421706 10.1371/journal.pbio.3000627 PMC7259792

[msaa288-B48] Martins E , Hansen T. 1997. Phylogenies and the comparative method: a general approach to incorporating phylogenetic information into the analysis of interspecific data. Am Nat. 149(4):646–667.

[msaa288-B49] Molina-Venegas R , Rodríguez M. 2017. Revisiting phylogenetic signal; strong or negligible impacts of polytomies and branch length information? BMC Evol Biol. 17(1):53. 28201989 10.1186/s12862-017-0898-y PMC5312541

[msaa288-B50] Münkemüller T , Lavergne S, Bzeznik B, Dray S, Jombart T, Schiffers K, Thuiller W. 2012. How to measure and test phylogenetic signal. Methods Ecol Evol. 3(4):743–756.

[msaa288-B51] Nehrt NL , Clark WT, Radivojac P, Hahn MW. 2011. Testing the ortholog conjecture with comparative functional genomic data from mammals. PLoS Comput Biol. 7(6):e1002073. 21695233 10.1371/journal.pcbi.1002073 PMC3111532

[msaa288-B52] Oakley TH , Gu Z, Abouheif E, Patel NH, Li WH. 2005. Comparative methods for the analysis of gene-expression evolution: an example using yeast functional genomic data. Mol Biol Evol. 22(1):40–50. 15356281 10.1093/molbev/msh257

[msaa288-B53] Oakley TH , Ostman B, Wilson AC. 2006. Repression and loss of gene expression outpaces activation and gain in recently duplicated fly genes. Proc Natl Acad Sci U S A. 103(31):11637–11641. 16864793 10.1073/pnas.0600750103 PMC1544222

[msaa288-B54] O’Meara BC , Ané C, Sanderson MJ, Wainwright PC. 2006. Testing for different rates of continuous trait evolution using likelihood. Evolution 60(5):922–933. 16817533

[msaa288-B55] Orme D. 2018. The caper package: comparative analysis of phylogenetics and evolution in R. Available from: https://cran.r-project.org/web/packages/caper/vignettes/caper.pdf.

[msaa288-B56] Pagel M. 1999. Inferring the historical patterns of biological evolution. Nature 401(6756):877–884. 10553904 10.1038/44766

[msaa288-B57] Panchin AY , Gelfand MS, Ramensky VE, Artamonova II. 2010. Asymmetric and non-uniform evolution of recently duplicated human genes. Biol Direct. 5(1):54. 20825637 10.1186/1745-6150-5-54 PMC2942815

[msaa288-B58] Paradis E , Claude J, Strimmer K. 2004. APE: analyses of phylogenetics and evolution in R language. Bioinformatics 20(2):289–290. 14734327 10.1093/bioinformatics/btg412

[msaa288-B59] Pegueroles C , Laurie S, Albà MM. 2013. Accelerated evolution after gene duplication: a time-dependent process affecting just one copy. Mol Biol Evol. 30(8):1830–1842. 23625888 10.1093/molbev/mst083

[msaa288-B60] Pennell MW , Eastman JM, Slater GJ, Brown JW, Uyeda JC, FitzJohn RG, Alfaro ME, Harmon LJ. 2014. Geiger v2.0: an expanded suite of methods for fitting macroevolutionary models to phylogenetic trees. Bioinformatics 30(15):2216–2218. 24728855 10.1093/bioinformatics/btu181

[msaa288-B61] Pich i Roselló O , Kondrashov FA. 2014. Long-term asymmetrical acceleration of protein evolution after gene duplication. Genome Biol Evol. 6(8):1949–1955. 25070510 10.1093/gbe/evu159 PMC4159008

[msaa288-B62] Purvis A , Rambaut A. 1995. Comparative analysis by independent contrasts (CAIC): an Apple Macintosh application for analysing comparative data. Comput Appl Biosci. 11(3):247–251. 7583692 10.1093/bioinformatics/11.3.247

[msaa288-B63] R Core Team. 2018. R: a language and environment for statistical computing. Vienna [Austria]: R Foundation for Statistical Computing. Available from: https://www.R-project.org/

[msaa288-B64] Revell LJ. 2012. Phytools: an R package for phylogenetic comparative biology (and other things). Methods Ecol Evol. 3(2):217–223.

[msaa288-B65] Rogozin IB , Managadze D, Shabalina SA, Koonin EV. 2014. Gene family level comparative analysis of gene expression in mammals validates the ortholog conjecture. Genome Biol Evol. 6(4):754–762. 24610837 10.1093/gbe/evu051 PMC4007545

[msaa288-B66] Rohlf FJ. 2001. Comparative methods for the analysis of continuous variables: geometric interpretations. Evolution 55(11):2143–2160. 11794776 10.1111/j.0014-3820.2001.tb00731.x

[msaa288-B67] Rohlfs RV , Nielsen R. 2015. Phylogenetic ANOVA: the expression variance and evolution model for quantitative trait evolution. Syst Biol. 64(5):695–708. 26169525 10.1093/sysbio/syv042 PMC4635652

[msaa288-B68] Sanderson MJ. 2002. Estimating absolute rates of molecular evolution and divergence times: a penalized likelihood approach. Mol Biol Evol. 19(1):101–109. 11752195 10.1093/oxfordjournals.molbev.a003974

[msaa288-B69] Scannell DR , Wolfe KH. 2007. A burst of protein sequence evolution and a prolonged period of asymmetric evolution follow gene duplication in yeast. Genome Res. 18(1):137–147. 18025270 10.1101/gr.6341207 PMC2134778

[msaa288-B70] Slowikowski K. 2018. Ggrepel: automatically position non-overlapping text labels with ‘ggplot2’. Available from: https://CRAN.R-project.org/package=ggrepel.

[msaa288-B71] Sonnhammer EL , Gabaldón T, Sousa da Silva AW, Martin M, Robinson-Rechavi M, Boeckmann B, Thomas PD, Dessimoz C, Quest for Orthologs Consortium. 2014. Big data and other challenges in the quest for orthologs. Bioinformatics 30(21):2993–2998. 25064571 10.1093/bioinformatics/btu492 PMC4201156

[msaa288-B72] Stamboulian M , Guerrero RF, Hahn MW, Radivojac P. 2020. The ortholog conjecture revisited: the value of orthologs and paralogs in function prediction. Bioinformatics 36(Suppl 1):i219–i226. 32657391 10.1093/bioinformatics/btaa468 PMC7355290

[msaa288-B73] Studer RA , Robinson-Rechavi M. 2009. How confident can we be that orthologs are similar, but paralogs differ? Trends Genet. 25(5):210–216. 19368988 10.1016/j.tig.2009.03.004

[msaa288-B74] Thomas GH , Freckleton RP, Székely T. 2006. Comparative analyses of the influence of developmental mode on phenotypic diversification rates in shorebirds. Proc R Soc B. 273(1594):1619–1624. 10.1098/rspb.2006.3488 PMC1634920 16769632

[msaa288-B75] Urbanek S. 2013. Png: read and write png images. Available from: https://CRAN.R-project.org/package=png.

[msaa288-B76] Uyeda JC , Harmon LJ. 2014. A novel Bayesian method for inferring and interpreting the dynamics of adaptive landscapes from phylogenetic comparative data. Syst Biol. 63(6):902–918. 25077513 10.1093/sysbio/syu057

[msaa288-B77] Uyeda JC , Pennell MW, Miller ET, Maia R, McClain CR. 2017. The evolution of energetic scaling across the vertebrate tree of life. Am Nat. 190(2):185–199. 28731792 10.1086/692326

[msaa288-B78] Warnes GR , Bolker B, Lumley T. 2018. Gtools: various R programming tools. Available from: https://CRAN.R-project.org/package=gtools.

[msaa288-B79] Wickham H. 2016. Ggplot2: elegant graphics for data analysis. New York: Springer. Available from: https://ggplot2.tidyverse.org.

[msaa288-B80] Wickham H. 2017. Tidyverse: easily install and load the ‘tidyverse’. Available from: https://CRAN.R-project.org/package=tidyverse.

[msaa288-B81] Wickham H. 2019. Stringr: simple, consistent wrappers for common string operations. Available from: https://CRAN.R-project.org/package=stringr.

[msaa288-B82] Wickham H , Francois R, Henry L, Müller K. 2017. dplyr: a grammar of data manipulation. Available from: https://CRAN.R-project.org/package=dplyr.

[msaa288-B83] Wilke CO. 2019. Cowplot: streamlined plot theme and plot annotations for ‘ggplot2’. Available from: https://CRAN.R-project.org/package=cowplot.

[msaa288-B84] Yanai I , Benjamin H, Shmoish M, Chalifa-Caspi V, Shklar M, Ophir R, Bar-Even A, Horn-Saban S, Safran M, Domany E, et al 2005. Genome-wide midrange transcription profiles reveal expression level relationships in human tissue specification. Bioinformatics 21(5):650–659. 15388519 10.1093/bioinformatics/bti042

