# Supplementary material for: Special Care Is Needed in Applying Phylogenetic Comparative Methods to Gene Trees with Speciation and Duplication Nodes
Source: Mol Biol Evol. 2020 Nov 10;38(4):1614–26. doi: 10.1093/molbev/msaa288 (PMC8042747; doi:10.1093/molbev/msaa288)
Supplement: msaa288_Supplementary_Data [file msaa288_supplementary_data.pdf]

Supporting Information

Table S1: Summary statistics of calibrated old duplication nodes for 8420 trees of Dunn et al. (2018).

Maximum age group in Million Years (My)	Count
296-500	2917
501-900	7548
901-3000	49
3001-10000	16
10001-11799977	9

Table S2: Analyses on multi optima OU trees.

Duplication age	Data	$\theta_{Speciation}$	$\theta_{Duplication}$	$\theta_{Duplication}$ / $\theta_{Speciation}$	*P*-value
Young	Empirical ($n_{Speciation}$= 2690; $n_{Duplication}$ = 842)	0.41	0.74	1.8	$8.6e^{-10}$
	Randomized τ ($n_{Speciation}$= 2690; $n_{Duplication}$ = 842)	0.53	0.55	1.03	0.97
	Randomized events ($n_{Speciation}$= 1872; $n_{Duplication}$ = 698)	0.50	0.53	1.06	0.75
Old	Empirical ($n_{Speciation}$= 4152; $n_{Duplication}$ = 847)	0.42	0.92	2.19	$2.4e^{-4}$
	Randomized τ ($n_{Speciation}$= 4152; $n_{Duplication}$ = 847)	0.54	$1.5e^{-11}$	$2.8e^{-11}$	$1.3e^{-07}$
	Randomized events ($n_{Speciation}$= 2081; $n_{Duplication}$ = 482)	0.51	0.66	1.29	0.73

Note: Median values of θ are shown. *P*-value from paired two-sided Wilcoxon test.

Table S3: Summary statistics on OUM trees, passing both the maximum likelihood and the Bayesian approaches with a posterior probability cutoff of ≥ 0.7.

Duplication Age	Proportions of regime shifts per branch		Paired two-sided Wilcoxon rank sum test	Regime shift rates (shifts/My)		Two-sided Wilcoxon rank test
	After speciation	After duplication		After speciation	After duplication	
Young	2.8%	8.3%	$8.3e^{-4}$	0.012	0.032	$6.7e^{-6}$
Old	0%	16.7%	$< 2.2e^{-16}$	0.012	0.0025	$< 2.2e^{-16}$

Note- Above analyses include 2779 speciation, 486 young, and 548 old duplication events. Values shown in the table indicate median values. The difference in proportions of regime shifts per branch after speciation events for two types of duplications is due to the different sets of trees used. Few trees shared both types of duplicates. Proportions of regime shifts per branch of events is estimated for each tree, and thus paired Wilcoxon test is used to compare the difference. A single gene tree can have one or many optima shift rate(s) for events, and thus two-sided Wilcoxon rank test was used for comparison.

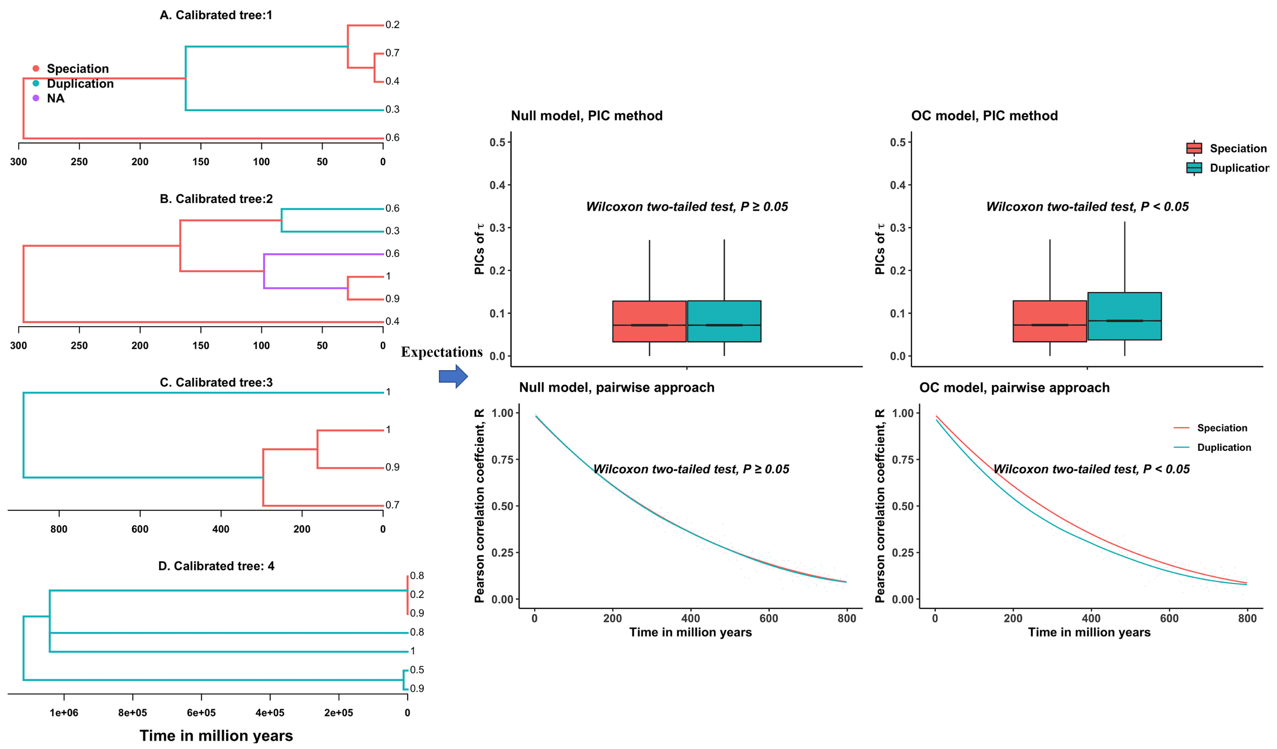

Fig. S1: Expectations from phylogenetic and pairwise comparison approaches under null and ortholog conjecture scenarios. PIC: Phylogenetic Independent Contrast, OC: Ortholog Conjecture. We present 4 time-calibrated gene trees of Dunn et al. (2018) as illustration. Branches are colored according to whether they arise from a speciation or a duplication node. Trees A and B are well calibrated, with the duplication ages are constrained by speciation ages, as shown by the time scales below each phylogeny. Trees C and D represent biased calibrated trees, where old duplication branches are inaccurately calibrated due to lack of age constraints. To evaluate the impacts of gene duplication and speciation events in trait evolution, pairwise comparisons do not rely on the branch lengths of a calibrated phylogeny, but phylogenetic methods do. If time calibration of old duplication nodes has no influence in the inference of phylogenetic approaches, we expect to obtain patterns under a null and OC scenarios as shown in the right part of the figure. This means that the phylogenetic contrasts or pairwise correlations of different events should be drawn from the same distribution under a null model, while the expectation differs under the OC model. We used 2 times higher rates of trait

evolution (τ here) following duplications than speciations (i.e. $\sigma^2_{duplication} = 2 * \sigma^2_{speciation}$) in this example for the OC model.

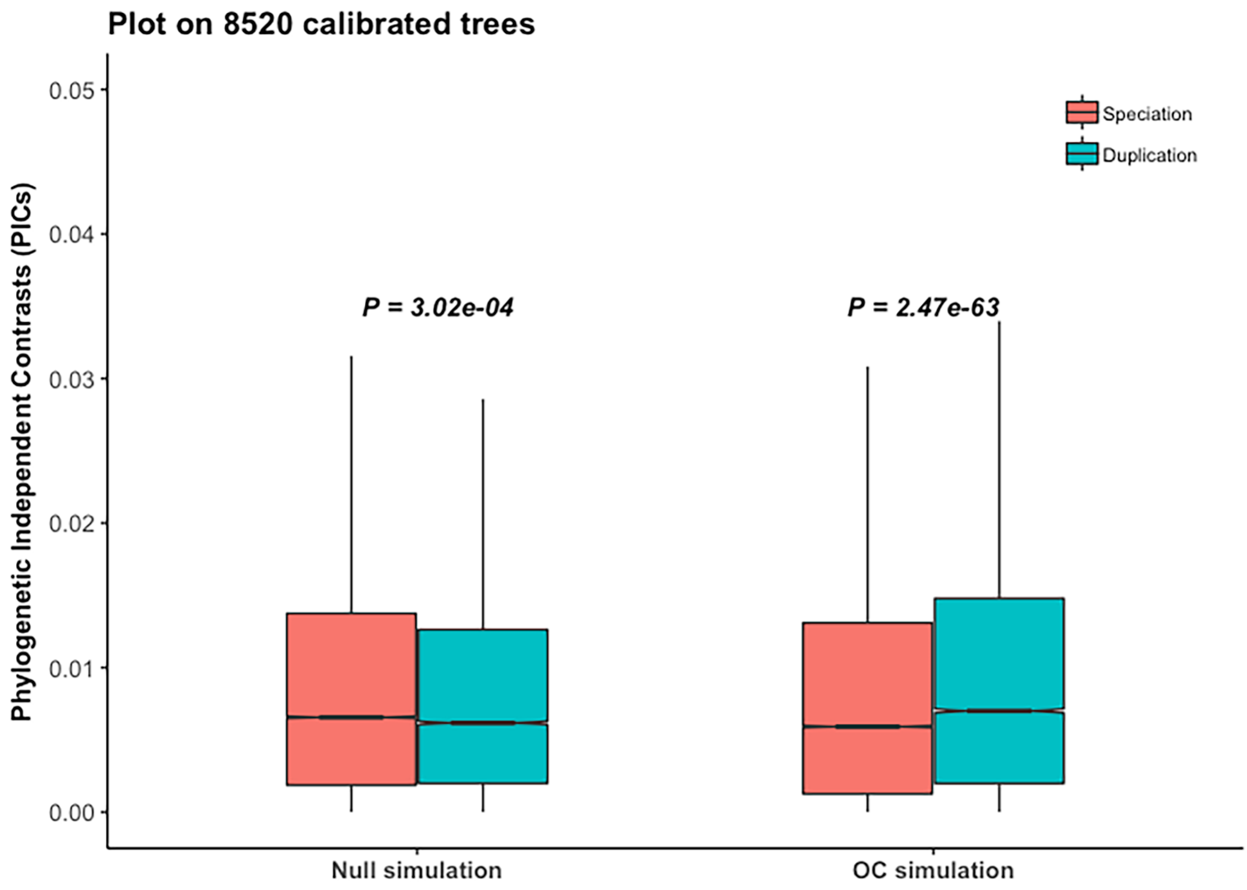

Fig. S2: Repeating simulations on all calibrated trees with different random seed number. *P* values are from Wilcoxon two-tailed tests. Simulations with different seed number did not change the trend of results as reported in Fig. 1A.

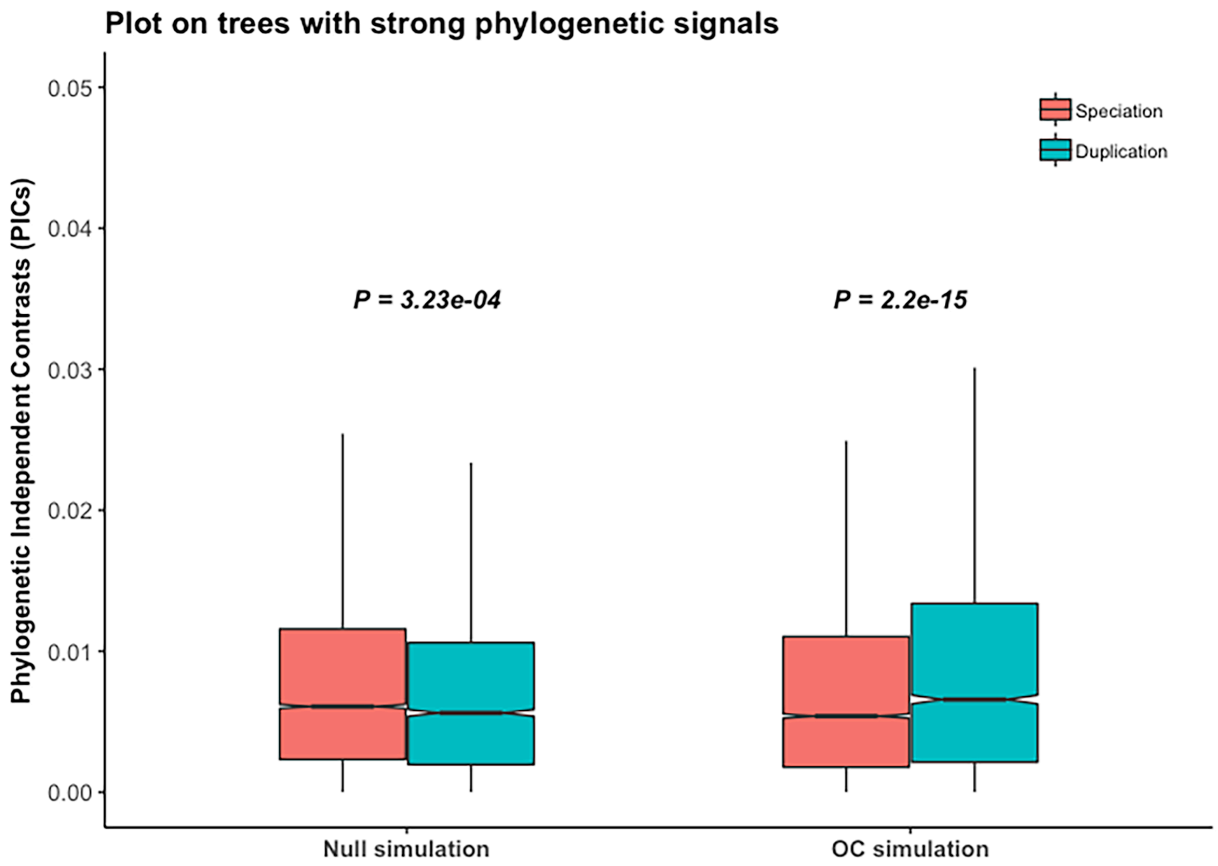

Fig. S3: Simulation analyses on 1135 trees with strong phylogenetic signals. *P* values are from Wilcoxon two-tailed tests. Dunn et al. used a cutoff of K > 0.551 to identify trees with strong phylogenetic signals. However, trees with higher K statistic can have corresponding *P* values which are non-significant. Considering both K statistic and *P* value, we found similar trends as was observed with 2082 trees.

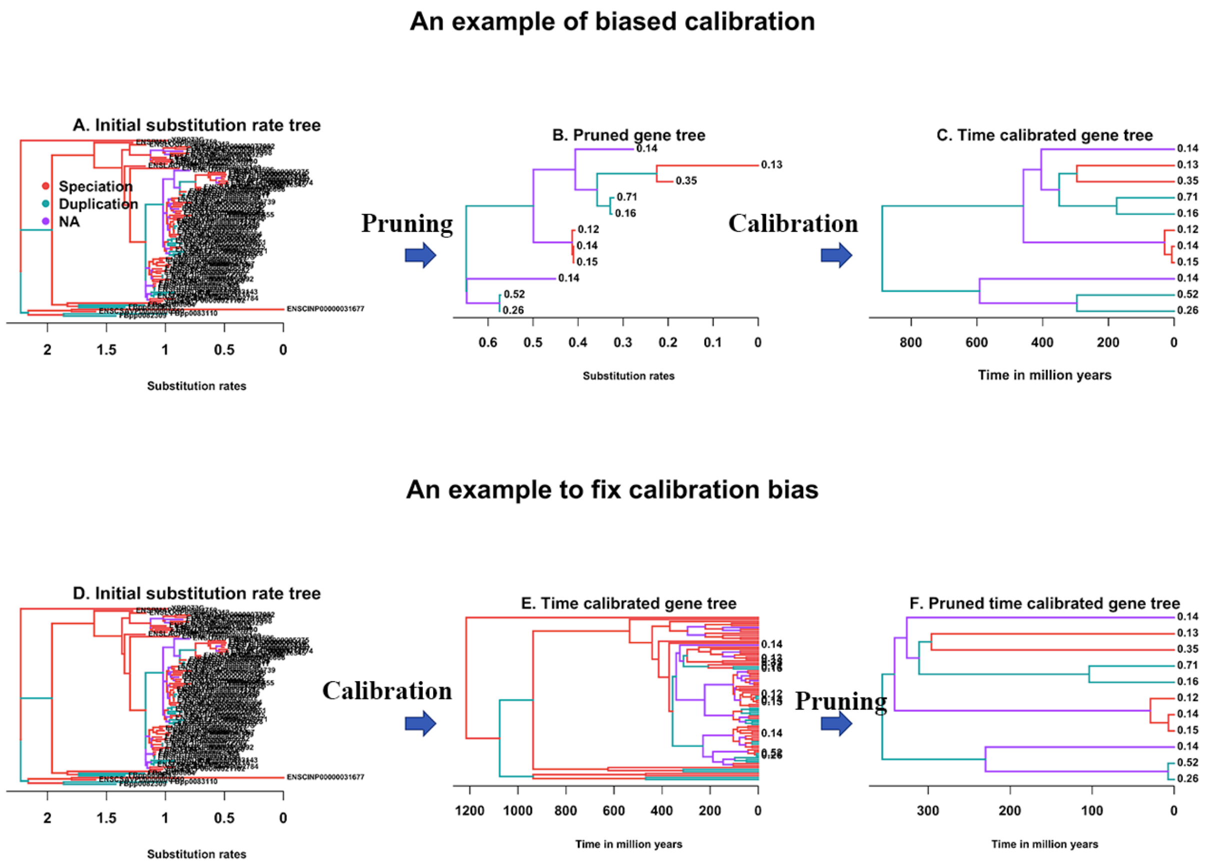

Fig. S4: Difference between time calibration approaches of Dunn et al. (2018) and of this study. In this example, we used the phylogeny of ACP1 gene. Branches are colored according to whether they arise from a speciation or a duplication node. The top panel (A-C) shows the steps used by Dunn et al. (2018), while the bottom panel (D-F) shows the steps used in this study. Gene trees obtained from Ensembl (Herrero et al. 2016) have branch lengths in substitutions per site. (A) and (D) are the same gene tree, where Dunn et al. (2018) edited few speciation events to 'NA' to pass the time calibration step. (B) The gene trees are pruned to species with available τ. (C) The pruned tree is time calibrated using speciation time points. Pruning before time calibration produces tree with many duplications, and NA nodes older to the oldest speciation nodes as in (B). This leads to using only 7 speciation time points for calibration. Due to unavailable age constraints on the old duplication nodes, the time scale of the phylogeny in (C) reaches 880 million years (My). When we performed time calibration

before pruning as in (E), we could use 32 speciation nodes for time calibration. This means that we could use many speciation nodes for time calibration, although τ data was unavailable for species at tips due to the choice of species in this study. Hence, the old duplication nodes are constrained by the age of speciation nodes older to them, and thus the maximum age is now of 356 My (F).

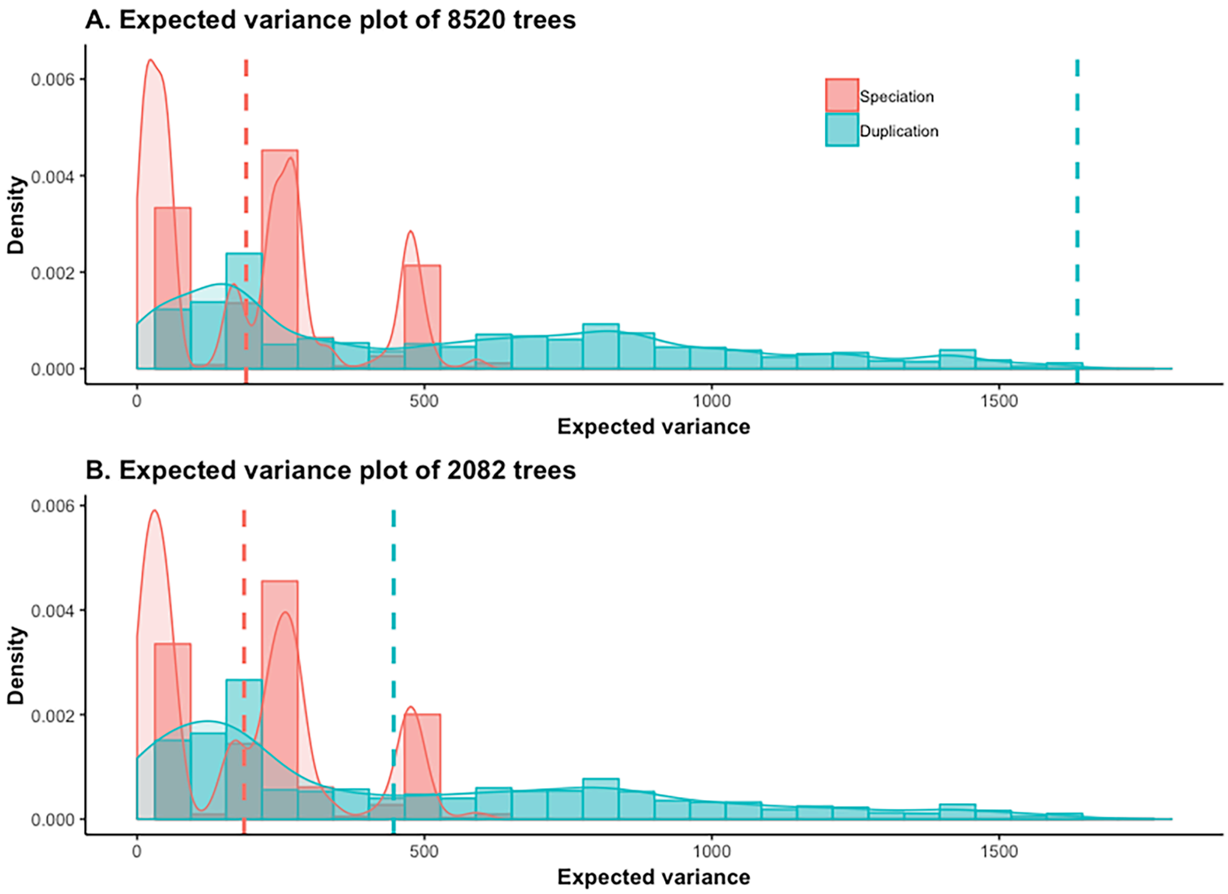

Fig. S5: Re-analyses of expected variances of calibrated trees considered by Dunn et al. The expected variance plots of (A) all 8520 calibrated trees, and (B) 2082 trees with strong phylogenetic signal. The dotted line represents the mean expected variance of the events. These plots show why duplication nodes preceding ancient speciation nodes can be problematic for PIC.

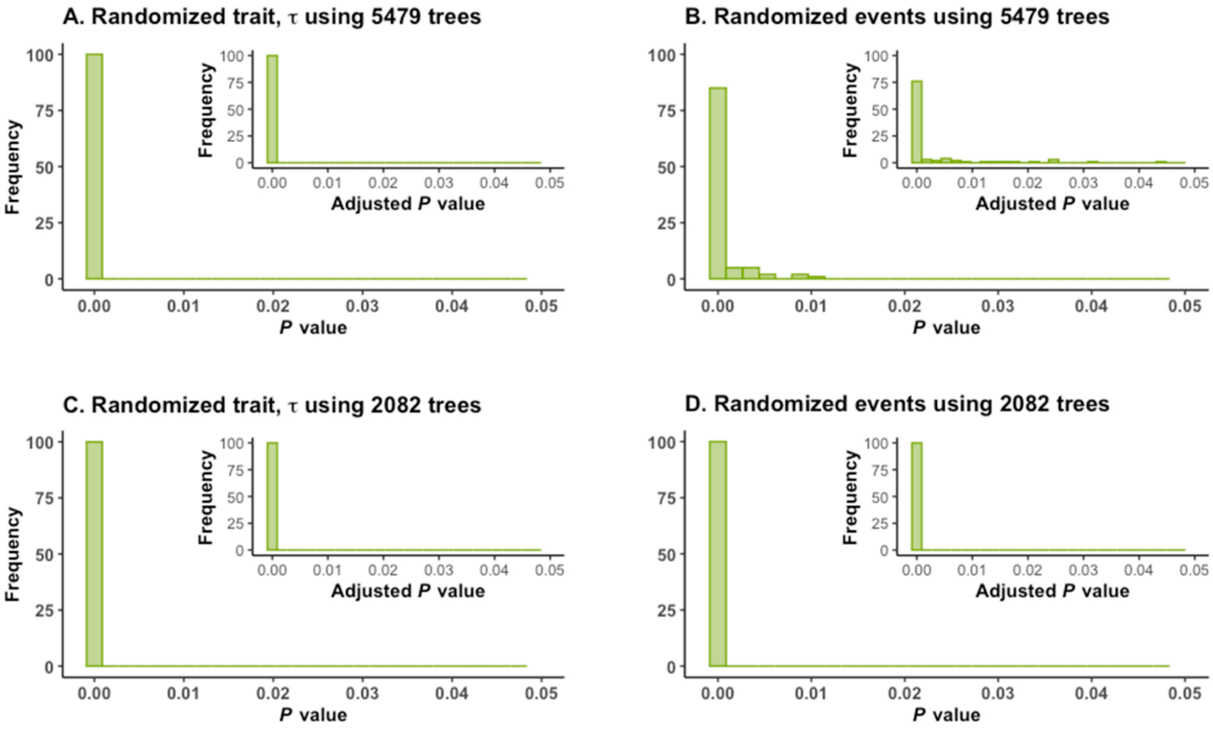

Fig. S6: *P* value distribution plots after 100 independent runs on each set of trees. Wilcoxon two-tailed test with 95% confidence interval was used to compare the speciation and duplication contrasts after randomization tests. (A) and (B) applied to trees with at least one speciation and one duplication event. (C) and (D) applied to trees with strong phylogenetic signal. (A) and (C) randomization of trait (τ) over the trees. (B) and (D) randomization of internal node events. The inset plots show *P* values adjusted with Benjamini-Hochberg (Benjamini and Yekutieli 2005; Hochberg and Benjamini 1990). Supporting our observations of Figs. 2A and 2C, all the plots confirm that the empirical result of Dunn et al. (2018) is not different from randomized test results.

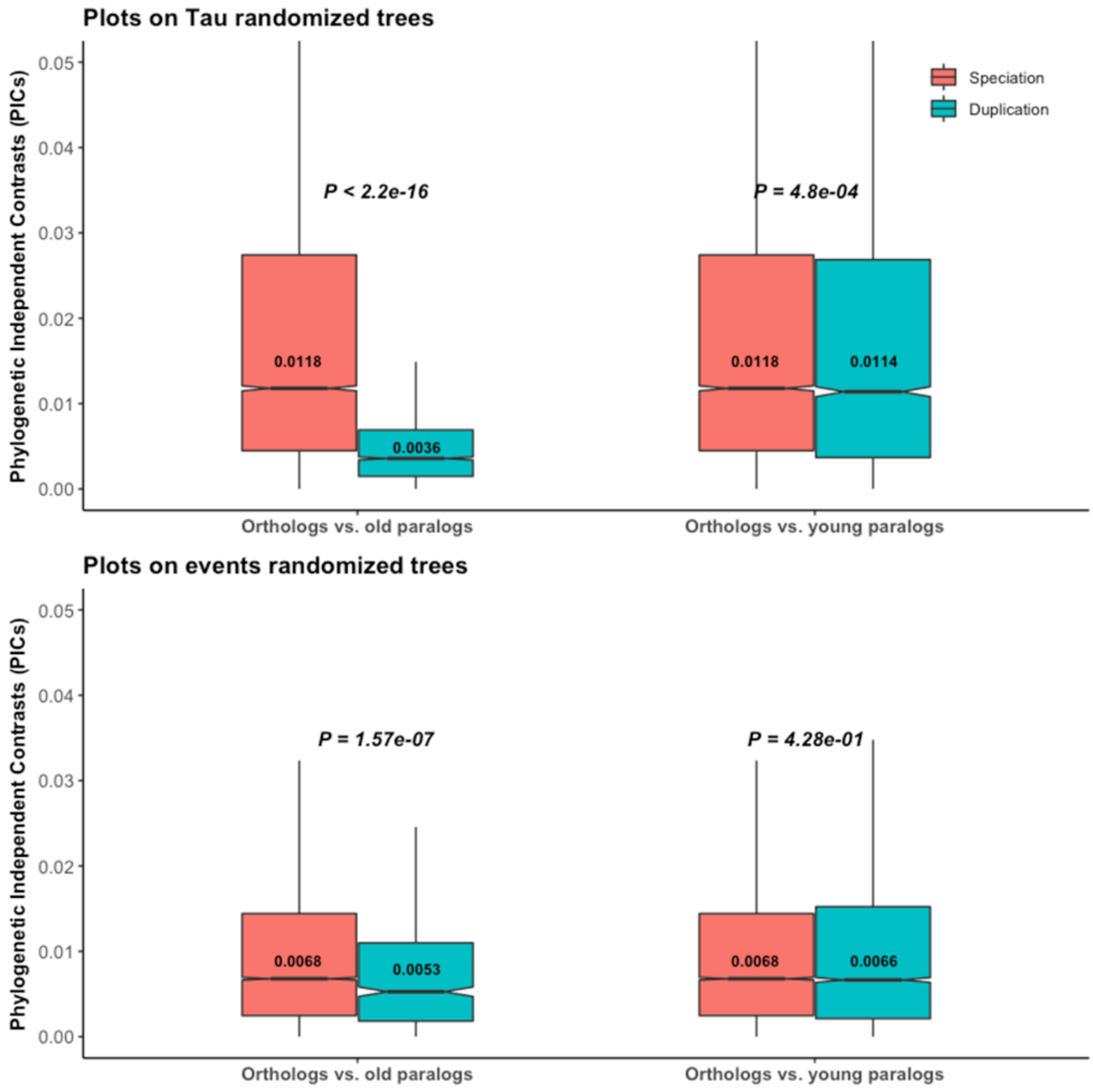

Fig. S7: The ortholog conjecture test after randomizations of contrasts standardized trees. *P* values are from Wilcoxon two-tailed tests. 'PICs': Phylogenetic Independent Contrasts. Values inside boxplots denote median PIC value of the corresponding event. (A-B) Plots after randomizing τ, and after randomizing events using the same trees as in Fig. 3.

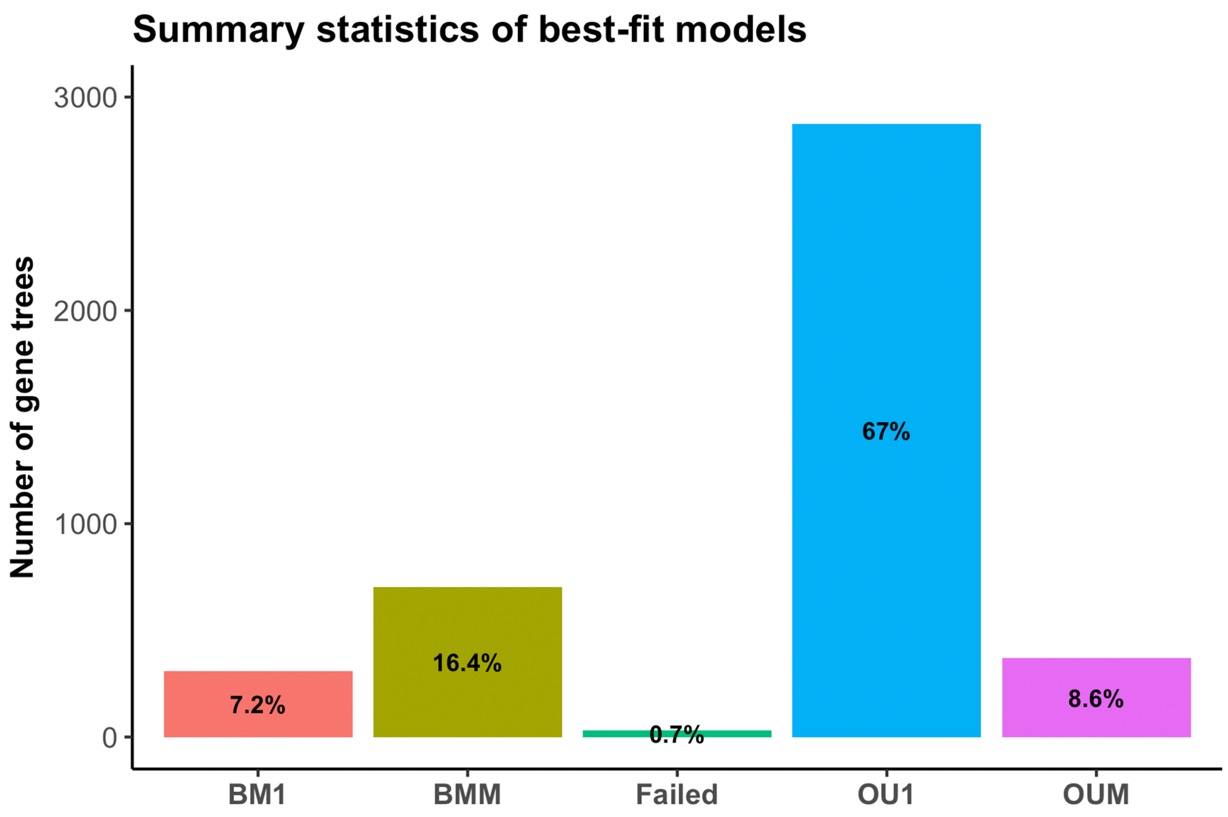

Fig. S8: Multivariate model fitting result using a maximum likelihood framework. BM1: Single rate Brownian; BMM: Multi rates Brownian; OU1: Single optimum Ornstein-Uhlenbeck; and OUM: Multi optima Ornstein-Uhlenbeck models.

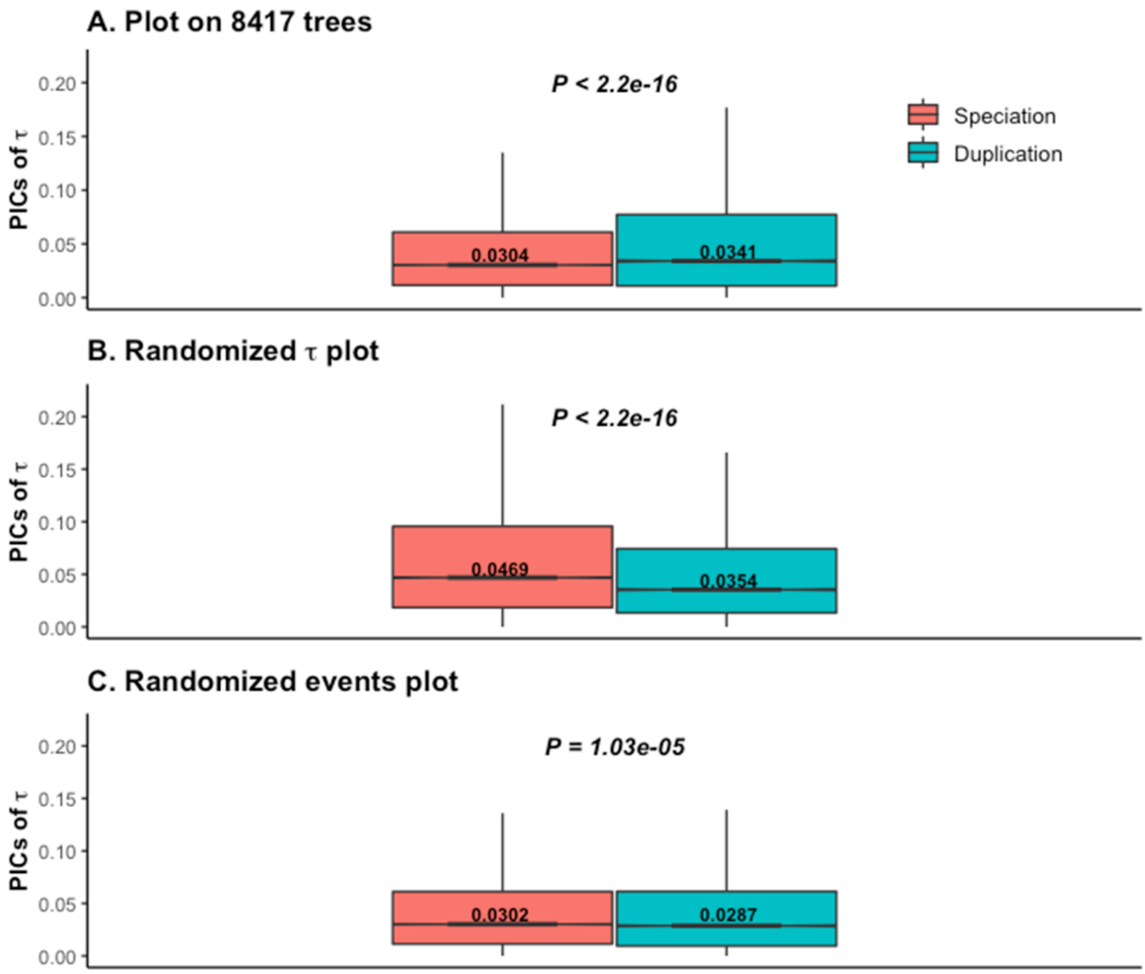

Fig. S9: The ortholog conjecture test for τ on calibrated trees of Dunn et al. *P* values are from Wilcoxon two-tailed tests. 'PICs': Phylogenetic Independent Contrasts. Values inside boxplots denote median PIC value of the corresponding event. (A) Using 8417 out of 8520 calibrated trees that passed diagnostic tests following branch length transformation. (B) Plot after randomizing τ, and (C) after randomizing events using the same branch transformed trees as in (A).

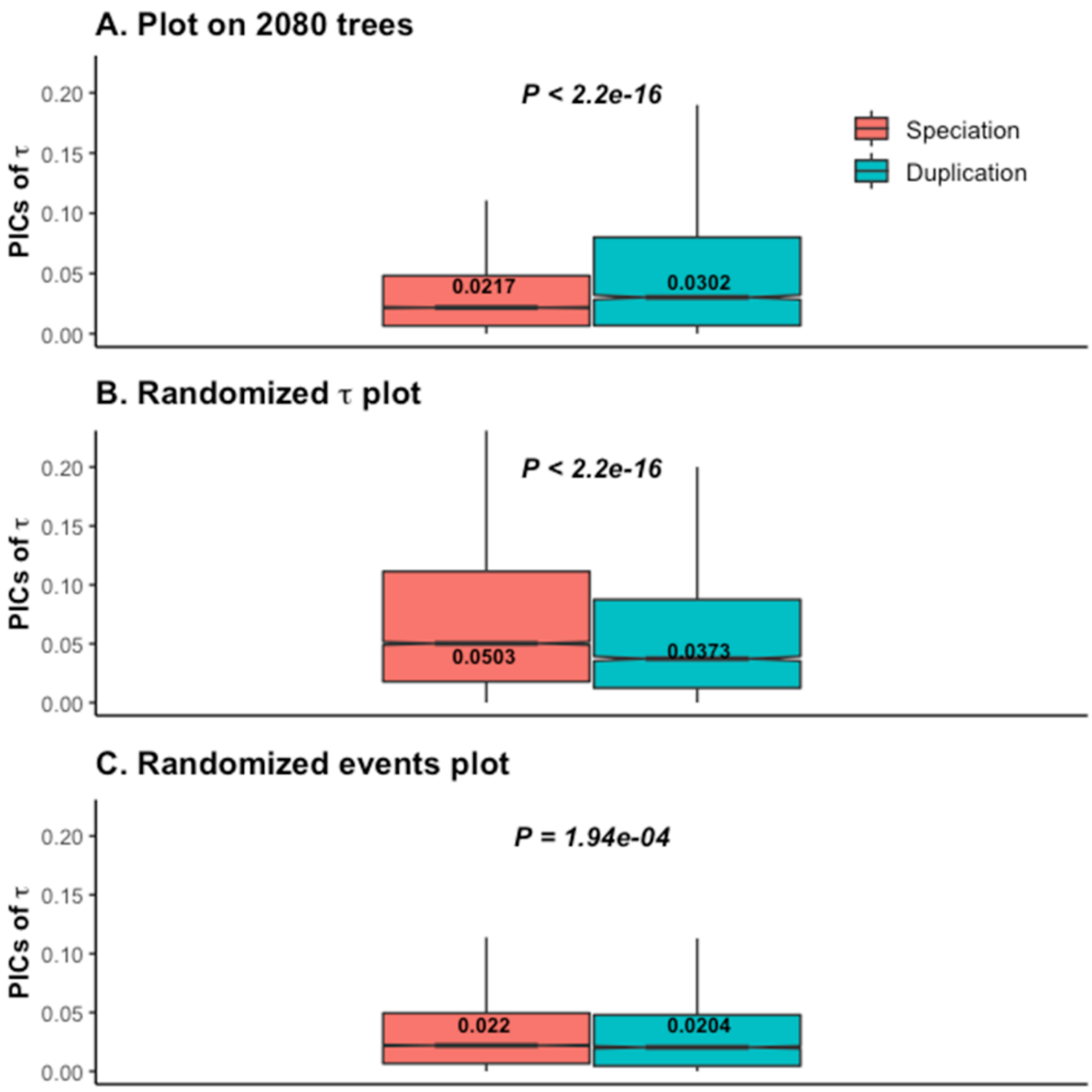

Fig. S10: The ortholog conjecture test for τ on branch transformed trees with strong phylogenetic signals. *P* value are from Wilcoxon two-tailed tests. 'PICs': Phylogenetic Independent Contrasts. Values inside boxplots denote median PIC value of the corresponding event. (A) using 2080 out of 2082 calibrated trees that passed diagnostic tests following branch length transformation. (B) Plot after randomizing τ, and (C) after randomizing events using the same branch transformed trees as in (A).
